# Antibody-Based Immunotherapies for the Treatment of Hematologic Malignancies

**DOI:** 10.3390/cancers16244181

**Published:** 2024-12-15

**Authors:** Justyna Jureczek, Krzysztof Kałwak, Piotr Dzięgiel

**Affiliations:** 1 Division of Histology and Embryology, Department of Human Morphology and Embryology, Faculty of Medicine, Wroclaw Medical University, 50-368 Wroclaw, Poland; piotr.dziegiel@umw.edu.pl; 2 Clinical Department of Paediatric Bone Marrow Transplantation, Oncology and Haematology, Faculty of Medicine, Wroclaw Medical University, 50-368 Wroclaw, Poland; krzysztof.kalwak@umw.edu.pl

**Keywords:** immunotherapy, monoclonal antibodies, hematologic malignancies, cancer, tumor, rituximab, multiple myeloma, acute lymphoblastic leukemia, lymphocytes, BiTe

## Abstract

Despite the great advancements in treatment strategies for hematological malignancies (HMs) over the years, their effective treatment remains challenging. Conventional treatment strategies are burdened with several serious drawbacks limiting their effectiveness and safety. Improved understanding of tumor immunobiology has provided novel anti-cancer strategies targeting selected immune response components. Currently, immunotherapy is counted as the fourth pillar of oncological treatment (together with surgery, chemo- and radiotherapy) and is becoming standard in the treatment regimen, alone or in combination therapy. Several categories of immunotherapies have been developed and are currently being assessed in clinical trials for the treatment of blood cancers, including immune checkpoint inhibitors, antigen-targeted antibodies, antibody–drug conjugates, tumor vaccines, and adoptive cell therapies. However, monoclonal antibodies (mAbs) and their derivatives have achieved the most notable clinical outcome so far. Since the approval of rituximab for treating B-cell malignancies, the availability of mAbs against tumor-specific surface molecules for clinical use has flourished. Antibody-based therapy has become one of the most successful strategies for immunotherapeutic cancer treatment in the last few decades, and many mAbs have already been introduced into standard treatment protocols for some hematologic malignancies. To further increase the efficacy of mAbs, they can be conjugated to radioisotopes or cytostatic drugs, so-called antibody–drug conjugates. Moreover, with the growing recognition of T-cell immunity’s role in cancer development, strategies aimed at enhancing T cell activation and inhibiting mechanisms that suppress T cell function are actively being developed. This review provides a comprehensive overview of the current status of immunotherapeutic strategies based on monoclonal antibodies and their derivatives, including antibody–drug conjugates, bispecific T-cell engagers, and checkpoint inhibitors, approved for the treatment of various HMs.

## Simple Summary

Hematologic malignancies (HMs), spanning an array of diverse neoplasms affecting the blood, bone marrow, and lymph nodes, pose a considerable clinical challenge. While conventional therapies, including chemotherapy, radiotherapy, and hematopoietic stem-cell transplantation (HSCT), are effective in most cases, the treatment resistance and side effects remain unresolved. Immunotherapy, the strategy to harness a patient’s immune system to fight against cancer, has revolutionized the landscape of available armamentarium over recent decades. In particular, impressive progress has been achieved in developing monoclonal antibodies and improving their functionality for clinical use in hemato-oncology. This review provides an overview of the current status of immunotherapeutic strategies based on monoclonal antibodies and their derivatives approved for the treatment of various HMs.

## 1. Introduction

The immunotherapeutic approach to cancer treatment has become of utmost interest in the past few decades. The importance of immunotherapy was first highlighted as 2013’s Breakthrough of the Year by *Science* [1] and was recently awarded the Nobel Prize for Physiology or Medicine in 2018 for the discovery of CTLA-4 (to James P. Allison) and PD1/PD-L1 (to Tasuku Honjo) [2]. While chemotherapy and radiation have been the dominant treatments of cancer for decades, their lack of an ability to distinguish between healthy and tumor cells has fueled the desire to create tumor-specific therapeutics. Currently, immunotherapy is counted as the fourth pillar of oncological treatment (together with surgery, chemo- and radiotherapy) and is becoming standard in the treatment regimen, alone or in combination therapy. There is a long history of efforts to harness a patient’s immune system as a tool in the battle with cancer. One of the earliest documented examples is the observation made by surgeon William B. Coley in 1893, who noted that post-surgical infections were linked to better outcomes in cancer patients [3]. However, these seminal observations had to wait a few centuries to become clinically relevant. The understanding of the complex involvement of the immune system in tumorigenesis and formulation of the cancer immunosurveillance theory were crucial for the development of cancer immunotherapeutic approaches [4]. The graft-versus-leukemia effect observed during allogeneic transplantation was the first clear example of the immune system’s powerful ability to target and eliminate cancer cells [5,6].

**Figure 1:**
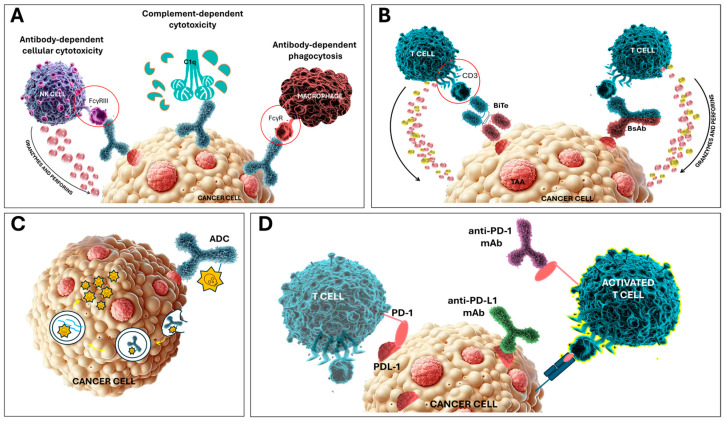
Mechanism of action of antibody-based therapeutics. Monoclonal antibodies (mAbs), can work indirectly through multiple mechanisms engaging elements of innate immunity, such as antibody-dependent cellular cytotoxicity, complement-mediated cytotoxicity, and antibody-dependent phagocytosis. Activated NK cells secrete granzyme and perforin molecules leading to tumor cell death. Complement-mediated cytotoxicity operates via the membrane attack complex, inhibiting receptor dimerization, triggering apoptosis, or affecting target cells by blocking ligand binding. Linking macrophages with tumor cells leads to antibody-dependent phagocytosis. Macrophages engulf antibody-coated tumor cells ultimately leading to the tumor cell’s degradation (**A**). Bispecific T-cell engagers recognize tumor-associated antigen and CD3 on T cells, simultaneously. After such cross-linkage, T cells are activated and secrete granzymes and perforins leading to tumor cell death. Bispecific engagers can be based on the full-size antibody structure or smaller derivatives (**B**). Once the antibody–drug conjugates (ADCs) bind to the tumor surface antigen, the antigen is internalized through endocytosis. The ADCs are then transported into the tumor cell and directed to the lysosome, where the cytotoxic payload is released. This payload induces apoptosis and can also kill nearby cancer cells via bystander effects (**C**). Immune checkpoint interaction takes place when PD-L1, found on the tumor cell, binds to PD-1, located on the T cell. This binding suppresses T-cell activation, thereby preventing the destruction of the tumor cell. Immune checkpoint inhibitors, such as anti-PD-1 and anti-PD-L1 antibodies, block their respective immune checkpoints, preventing PD-L1 from binding to PD-1. This action restores T-cell activation, leading to the destruction of tumor cells (**D**).

In the meantime, monoclonal antibodies (mAbs) have evolved fiercely from research tools to powerful therapeutics over the years [7,8]. Since the first mAb drug was approved (muromonab, OKT3) in 1986 to treat transplant rejection, and shortly later rituximab, the first mAb for the treatment of cancer, over 160 antibody therapeutics have come to be marketed to date [9,10]. Moreover, the number of protein therapeutics entering clinical development, including antibodies, antibody fragments, bispecifics, Fc-fusion proteins, and antibody–drug conjugates, is expected to further grow, mainly due to technological advancements in their production and high success rates in the clinic [11,12]. The massive progress in antibody research became possible thanks to murine hybridoma technology, which was later followed by humanization techniques to create therapeutic antibodies with reduced immunogenicity [13,14]. Advances in display technologies and the use of transgenic animals have further expanded the potential to generate antibodies with fully human sequences [15,16,17]. Antibodies can target cell surface receptors to either stimulate or block signaling, trigger antibody-dependent cell-mediated cytotoxicity, or initiate complement-dependent cytotoxicity, as depicted in Figure 1. Their mechanisms of action include direct tumor cell killing through receptor blockade or activation, inducing apoptosis, drug delivery, immune-mediated killing, regulating T-cell function, and impacting the tumor vasculature and stroma [16,17]. 

Hematologic malignancies refer to neoplasms originating from the hematopoietic and lymphoid tissues representing a broad spectrum of diseases affecting blood, bone marrow, and lymph nodes. In general, they are characterized by clonality and uncontrolled cell proliferation, the suppression of normal hematopoiesis, and infiltration into other tissues [18,19,20,21]. However, besides some shared characteristics, these conditions display significant differences in genetic causes and clinical course, which implies the need for variable management strategies [22,23].

In 2001, the World Health Organization (WHO) introduced a new consensus approach to the classification of hematologic tumors [24]. This modern approach was developed by expert hematopathologists to address the historical inconsistency in classification methods and has helped to build an international consensus, and has been revised and updated over the years. WHO classification has enabled the defining of multiple entities based on the principles of the REAL classification, incorporating morphology, immunophenotyping, genomics, and clinical features. Throughout the scientific literature, HMs have been mainly categorized as leukemia, lymphoma, and plasma cell neoplasms, including subtypes like Hodgkin versus non-Hodgkin lymphoma, acute versus chronic, and lymphatic versus myeloid leukemia [25]. The prevalence of hematologic malignancies is on the rise globally, making them one of the most common and lethal types of tumors. It is crucial to identify effective treatment approaches to tackle this increasing problem [26]. Despite major improvements in the management of HMs and the observed prolonged follow-up of survivors, conventional therapies have limited efficacy and relapses with high mortality rates are still frequent [27].

The landscape in the management of HMs is rapidly changing with the introduction of novel therapies and their combinations. Below we present an overview of the most notable advancements in immunotherapeutics based on mAbs approved for the treatment of HMs in recent years.

## 2. Immunotherapeutics in Hematology Malignancies

### 2.1. Monoclonal Antibodies

Monoclonal antibodies (mAbs) were among the pioneering types of immunotherapy approved for anti-cancer treatment and hold a vital and increasingly important role in current treatment regimens. Notably, rituximab, the anti-CD20 mAb was the first mAb implemented in oncology and remains the most commonly applied [28]. The approval of rituximab in 1997 revolutionized the treatment of B-cell lymphomas and was an important milestone in the advent of newer immunotherapeutic approaches.

Rituximab is a chimeric human–mouse mAb that targets CD20, a common B-cell surface marker, and facilitates the specific depletion of these cells [29]. The CD20 antigen is highly expressed on the surface of B cells as well as the majority of B-cell lymphomas but is absent from hematopoietic stem cells, differentiated plasma cells, and other healthy tissues, making it a suitable target for the efficient induction of effector mechanisms that mediate the specific depletion of B cells [30]. Antibody-dependent cell-mediated cytotoxicity and complement-dependent cytotoxicity are key mechanisms contributing to rituximab-mediated B-cell depletion [31]. Since mature plasma cells and B-cell progenitors do not express CD20, targeting and depleting B cells at these intermediate stages of development typically does not result in lasting side effects [32,33]. With over 20 years of clinical experience using rituximab, it has proven to be highly effective and safe, despite initial doubts. Rituximab has significantly improved outcomes in B-cell malignancies, including follicular lymphoma (FL), diffuse large B-cell lymphoma (DLBCL), and chronic lymphocytic leukemia (CLL) [34,35]. Moreover, its high efficiency in B-cell depletion was found to be of central interest in the treatment of EBV-PTLD in the post-allogeneic hematopoietic stem-cell transplantation (alloHSCT) setting [36,37].

Based on this pioneering success of rituximab in clinical practice, over the last decade, we can observe the high expansion of novel mAbs approved in the hemato-oncology field, as summarized in Table 1. Ofatumumab is a second-generation, fully humanized anti-CD20 mAb that targets an alternative epitope than rituximab and binds its target with higher affinity. It was approved by the FDA for treating CLL refractory to fludarabine and alemtuzumab in 2009 as monotherapy, and later in 2014, for use in combination with chlorambucil [38,39]. However, it was discontinued in all non-US markets in 2019 due to economic reasons, and its use in those regions is restricted to compassionate access only. Nevertheless, it was recently approved with a new indication in treating relapsing forms of multiple sclerosis in adults [40]. Obinutuzumab, another second-generation anti-CD20 mAb, was approved by the FDA in 2013 for use with chlorambucil in CLL treatment and in 2016 for use with bendamustine in treating relapsed or refractory FL (R/R FL) [41,42]. Obinutuzumab (GA101) is a humanized, glycoengineered IgG1 type II mAb targeting the epitope on the large extracellular loop of CD20, which partially overlaps with the rituximab epitope [43]. Recent findings from a phase III trial have shown the superior effectivity of obinutuzumab over rituximab when included in immunochemotherapy for first-line treatment of FL [44]. Conversely, no advantage over rituximab was demonstrated in DLBCL [45]. However, the matter of the doses applied of both mAbs in comparable studies is open to discussion [35]. Several other clinical trials with obinutuzumab are in progress, including those comparing its effectivity over rituximab in other CD20-positive malignancies, like B-cell acute lymphoblastic leukemia (B-ALL) (NCT04920968) and mantle cell lymphoma [46,47,48]. Moreover, the first rituximab biosimilars, CT-P10 (Truxima^®^; developed by Celltrion) and GP2013 (Rixathon^®^; developed by Sandoz), were approved by the EMA in 2017 [49].

In addition to CD20, novel antigenic targets have been explored lately, and CD19, CD22, CD33, CD38, and SLAMF7 belong to the group most extensively studied in HM applications [50]. Elotuzumab, an anti-SLAMF7 mAb, was the first mAb approved in the treatment of MM and established a paradigm shift in immunotherapies for MM [51]. It demonstrated significantly improved 1-year and 2-year progression-free survival (PFS) rates of 68% and 41%, respectively, when combined with lenalidomide and dexamethasone, compared to these agents alone, achieving an overall response rate (ORR) of 79% [27]. It was approved by the FDA in 2015 in combination with lenalidomide and dexamethasone for the R/R MM treatment. New elotuzumab-containing combinations are in clinical trials [52].

CD38 is a transmembrane glycoprotein widely expressed on MM cells, and other mAbs targeting this attractive antigen are in active research [53]. Daratumumab, an anti-CD38 mAb, received FDA approval for treating relapsed or refractory multiple myeloma (R/R MM) in 2016, in combination with lenalidomide and dexamethasone, or bortezomib and dexamethasone, for the treatment of patients with MM who have received at least one prior therapy [54]. Another CD38 mAb, isatuximab was approved in 2020, in combination with pomalidomide and dexamethasone for the treatment of adult patients with MM who have received more than two prior therapies, and, in 2021, for combination with carfilzomib and dexamethasone, for the treatment of adult patients with R/R MM who have received one to three prior lines of therapy [55,56]. Moreover, in September 2024, the FDA approved isatuximab with bortezomib, lenalidomide, and dexamethasone for adults with newly diagnosed multiple myeloma who are not eligible for autoHSCT, based on results from the phase III IMROZ trial [57].

The latest approval of tafasitamab (Monjuvi^®^), a humanized, anti-CD19, Fc-modified mAb, was embraced enthusiastically among clinicians. DLBCL is the most prevalent form of non-Hodgkin lymphoma, comprising 25–45% of new lymphoma cases annually [26]. The introduction of rituximab in combination with cyclophosphamide, doxorubicin, prednisone, and vincristine (R-CHOP) as the standard first-line immunotherapy has significantly improved patient outcomes with this entity. However, 30–40% of patients still experience relapse or are resistant to this initial treatment [58]. For these relapsed or refractory patients, effective and well-tolerated alternative treatments remain scarce, resulting in a poor prognosis. On 31 July 2020, the FDA granted an accelerated approval for Monjuvi^®^ in combination with lenalidomide for adult patients with R/R DLBCL who are not eligible for autologous stem-cell transplant [59]. The approval was granted based on findings from the single-arm, phase II L-MIND study (NCT02399085), which enrolled 81 participants. Patients were treated with tafasitamab at a dose of 12 mg/kg intravenously in combination with lenalidomide for up to twelve 28-day cycles, followed by tafasitamab monotherapy. The primary endpoint was to assess the best ORR and duration of response. Among the 80 patients who received the tafasitamab–lenalidomide combination, 60% achieved an objective response, 43% had a complete response, and 18% had a partial response. In 71 DLBCL patients, the best ORR was 55%, with 37% achieving complete and 18% partial responses. The median duration of response was 21.7 months [60,61,62,63]. Numerous novel mAbs are being investigated in clinical trials and the marketing authorization of mAbs is an actively growing field, nicely reviewed annually by the “Antibodies to Watch” series [9,10].

### 2.2. Bispecific Antibodies and T-Cell Engagers

The improvement of mAb-based immunotherapy brought about the idea of bispecific Abs (BsAbs), which can bind two different target antigens or two different epitopes on the same antigen simultaneously [64,65]. Various designs of BsAbs have been developed over time differing in format and size [66]. The most widely used BsAb format is based on selective recruitment of potent effector cells of the immune system toward tumor cells. This is achieved by simultaneously binding a tumor-associated antigen (TAA) on tumor cells, and an activating receptor on immune effector cells, such as a CD3 molecule on the effector T lymphocytes in the case of the class of T-cell engagers [67,68]. Such BsAb-mediated cross-linkage results in the activation of the effector cells in a non-MHC-restricted manner, leading to efficient tumor cell eradication [69]. Another discrimination factor within bispecifics is the presence or absence of an Fc region. The interaction between Fc domains and their receptors on various types of immune effector cells such as natural killer (NK) cells, monocytes, and macrophages, is capable of inducing antibody-dependent cell-mediated cytotoxicity. The Fc domain can also bind complement to elicit complement-dependent cytotoxicity, leading to the unnecessary non-specific immune response during bsAb treatment [70]. Hence, the shorter fragments (without Fc portions) are currently more explored, usually consisting of single-chain variable regions (scFvs) of two parenting mAbs connected by a flexible linker [71,72]. Of these novel bispecific platforms, the bispecific T-cell engagers (BiTEs) have demonstrated the greatest clinical relevance so far, with Blinatumomab being the standout example [73].

Blinatumomab is a CD19xCD3 BiTE approved for the treatment of patients with Philadelphia chromosome-negative precursor B-cell acute lymphoblastic leukemia (B-ALL) [74]. Due to its small size (~55 kDa), blinatumomab can reach within close proximity to a T cell and target-cell epitopes, which enables highly specific T-cell activation [75]. The human CD19 antigen is a transmembrane glycoprotein from the immunoglobulin superfamily. It is specifically expressed on normal and neoplastic B cells, as well as on follicular dendritic cells [76]. The surface expression of CD19 begins during B-cell lymphopoiesis, around the time of immunoglobulin gene rearrangement, and its expression is tightly regulated throughout B-cell development and maturation, ceasing during terminal plasma cell differentiation [77]. In mature B cells, CD19 expression is three times higher than in immature B cells. It plays a critical role in regulating B-cell signaling thresholds by modulating both B-cell receptor-dependent and independent pathways [78]. The fact that CD19 is expressed by a wide range of B-lymphoid malignancies, but not by hematopoietic stem cells and pro-B cells, makes it an attractive target for antibody-mediated therapy [79]. Blinatumomab has demonstrated significant efficacy in adult and pediatric patients with R/R B-ALL and patients with measurable residual disease (MRD). Clinical studies showed that over 70% of MRD-positive patients achieved MRD negativity, which correlates with better long-term outcomes and improved overall survival (OS) [80]. Based on its acceptable safety profile and high response rates, blinatumomab was approved by the FDA for treating R/R B-ALL in both adult and pediatric patients in 2014 [73]. Since then, the amount of clinical data on blinatumomab has continuously increased. The NEUF retrospective observational study investigated the safety and effectiveness of blinatumomab in adult patients with R/R B-ALL in real-world clinical settings. A total of 140 R/R B-ALL patients were included, with 106 being Ph^−^ and 34 Ph^+^. These real-world data support the efficacy results seen in randomized clinical trials of blinatumomab [81]. Moreover, recent data show the encouraging efficacy of blinatumomab in certain ALL contexts, including its use as a frontline therapy, as a bridge to transplantation, and in “chemotherapy-free” combination treatment regimens, both in adults and pediatric populations [82,83,84,85]. In a phase III randomized clinical trial, pediatric patients with high-risk, first-relapse B-ALL received blinatumomab as a consolidation therapy before undergoing alloHSCT. This treatment led to improved event-free survival (EFS) and higher rates of minimal residual disease remission compared to chemotherapy. The EFS benefit was seen across all patient subgroups, including those with extramedullary disease and very early relapse (within 18 months) [86]. A longer follow-up revealed significantly better OS in patients treated with blinatumomab compared to chemotherapy, regardless of their MRD status before treatment [87]. Furthermore, the latest post hoc analysis of this study shows that children with high-risk, first-relapse B-ALL who received blinatumomab as the third consolidation therapy before alloHSCT had improved 2-year OS and EFS estimates compared to those who received HC3 (dexamethasone, vincristine, daunorubicin, methotrexate, ifosfamide, and PEG-asparaginase), regardless of whether they were treated with total body irradiation plus etoposide or chemoconditioning [88]. However, the sample size and the post hoc nature of the analysis may have limited applicability, hence the ongoing FORUM study, which includes over 1700 registered children with ALL, is expected to provide more comprehensive data on the benefits of using blinatumomab before alloHSCT in real-world settings [89].

Building on its successful outcomes, additional BsAbs have been developed over time and studied in clinical trials, but we have only seen a real breakthrough in the last two years. Six novel BsAbs for hemato-oncology applications achieved FDA approval in the last two years, as summarized in Table 2. The CD20xCD3 BsAbs mosunetuzumab, epcoritamab, and glofitamab have been granted accelerated FDA approval for certain subtypes of non-Hodgkin lymphoma due to their significant efficacy in clinical trials [90].

BCMA is currently the main target for immunotherapies in MM due to its highly restricted expression, minimizing the potential of off-target effects. It is predominantly expressed on differentiated plasma cells and plasmablasts, while not expressed on naïve B cells, hematopoietic stem cells, or normal non-hematologic tissues [91,92]. Teclistamab, approved in 2022, is a novel T-cell-redirecting BsAb targeting BCMA and CD3. It was evaluated in the phase I/II MajesTEC trial involving heavily pretreated patients with R/R MM [93]. The ORR achieved 65%, with a median PFS of 11.3 months [94]. Another BsAb-targeting BCMA and CD3 is elranatamab. It was approved in 2023 for adult patients with R/R MM who have undergone at least four prior therapies, including a proteasome inhibitor, an immunomodulatory agent, and an anti-CD38 mAb, showing promising results in clinical trials [95]. In the MagnetisMM-1 trial, involving heavily pretreated R/R MM patients, the median PFS was 11.8 months, and the OS was 21.2 months. At a 12-month follow-up, the ORR was 63.6%, with 38.2% achieving a CR. Cytopenias and cytokine release syndrome (CRS) were reported, though no dose-limiting toxicities occurred. In the phase II MagnetisMM-3 trial, patients responding to weekly elranatamab were transitioned to bi-weekly dosing to enhance tolerability, resulting in a 61.0% ORR and a 35% CR rate. At 15 months, the duration of response (DOR) rate was 71.5%, PFS was 50.9%, and OS was 56.7%, with infections, CRS, anemia, and neutropenia being common adverse events [96,97,98].

BsAbs targeting myeloma cell antigens beyond BCMA have demonstrated potential in heavily pretreated R/R MM patients, including those who have previously received BCMA-targeted treatments [99]. The unique example in this class is talquetamab. Besides binding CD3 on T cells with one arm, the second one specifically binds to G-protein-coupled receptor family C, group 5, member D (GPRC5D), an orphan receptor present on the surface of normal plasma cells and myeloma cell lines [100]. In the MonumenTAL-1 phase I study, talquetamab showed promising results in heavily pretreated R/R MM patients, with an ORR of 70% for intravenous and 64% for subcutaneous administration, and a median DOR of 10.2 and 7.8 months, respectively. CRS was reported in 77–80% of patients. Follow-up data confirmed a sustained ORR of 73% with a median DOR over one year. In the phase II MonumenTAL-2 study, the combination of talquetamab and pomalidomide demonstrated efficacy and tolerability in the same patient population [101,102].

### 2.3. Antibody–Drug Conjugates

Immunoconjugates, or antibody–drug conjugates (ADCs), are a group of mAbs attached to a given cytotoxic or radioactive payload working as sophisticated delivery systems. The specific binding of Ab with its antigen enhances the targeted delivery to the tumor site, increasing the efficacy of the small molecule while reducing side effects and non-specific toxicity to non-target tissues [103]. The Ab can also be linked to a radionuclide to more precise targeted radiotherapy at the tumor site [104]. The initial enthusiasm for these targeted drug delivery systems surged with the approval of gemtuzumab ozogamicin in 2000 but waned following its withdrawal, due to significant hepatotoxicity in 2010 [105]. However, updated data on the clinical effectiveness and safety of gemtuzumab ozogamicin given in a fractionated dosing regimen resulted in its re-approval in 2017 for both newly diagnosed and R/R acute myeloid leukemia (AML) cases [106]. Technological advancements, including a better selection of cytotoxic agents and the use of smaller conjugates, have significantly increased the potential clinical benefits of ADCs [107]. Several ADCs have been designed and applied for clinical use in hematologic malignancies and their targets include CD19, CD20, CD22, CD30, CD33, CD37, CD79, CD123, and BCMA [108,109].

Currently, there are thirteen FDA-approved ADCs in total, and seven of them with hemato-oncology applications (as summarized in Table 3). In addition, more than 100 ADC molecules are at different stages of clinical trials.

Brentuximab vedotin (BV), an anti-CD30 Ab conjugated to the microtubule inhibitor monomethyl auristatin E (MMAE), has demonstrated efficacy in treating relapsed/refractory Hodgkin lymphoma (R/R HL) and systemic anaplastic large-cell lymphoma. These data led to its FDA approval for cancer treatment in 2011 and for post-autologous HSCT consolidation in 2015 [110]. In 2018, based on results from the phase III ECHELON-1 clinical trial, the FDA expanded the approval of brentuximab vedotin to include the treatment of adult patients with previously untreated stage III or IV classical Hodgkin lymphoma (cHL) in combination with AVD (doxorubicin, vinblastine, and dacarbazine) [111]. Lately, the update after 5 years of follow-up was published, in which BV+AVD (brentuximab vedotin, doxorubicin, vinblastine, and dacarbazine) demonstrated significant and lasting improvements in PFS compared to ABVD (doxorubicin, bleomycin, vinblastine, and dacarbazine), regardless of PET-2 status, while maintaining a consistent safety profile [112]. Furthermore, the BV was tested in clinical trials with newly diagnosed advanced-stage cHL led by the German Hodgkin Study Group, investigating its use in combination with chemotherapy. In the randomized, multicenter, open-label, phase III, HD21 clinical trial the BrECADD regimen (BV, etoposide, cyclophosphamide, doxorubicin, dacarbazine, and dexamethasone) was compared with the escalated BEACOPP regimen (bleomycin, etoposide, doxorubicin, vincristine, prednisone, and procarbazine) in a PET2-guided treatment approach. The BrECADD regimen demonstrated superior PFS compared to eBEACOPP, with 4-year PFS rates of 94.3% versus 90.9%, respectively. OS rates were similar between the two regimens, at approximately 98.5%. Further, BrECADD showed a more favorable safety profile, with significantly fewer treatment-related morbidity events (42% vs. 59%). It also preserved gonadal function better than eBEACOPP, with higher recovery rates of fertility markers in both men and women. These findings suggest BrECADD is more effective and better tolerated, positioning it as a strong alternative to eBEACOPP for advanced-stage classical Hodgkin lymphoma [113].

In 2017, inotuzumab ozogamicin (INO), a humanized anti-CD22 mAb linked to the cytotoxic antibiotic calicheamicin, was approved as a monotherapy for the treatment of CD22-positive B-ALL [114]. Within the INO-VATE trial, inotuzumab ozogamicin demonstrated significantly superior efficacy compared to the standard of care (SC) in patients with R/R B-ALL. The CR and CR with incomplete hematologic recovery (CRi) rates in the first 218 randomized patients, were 80.7% in the inotuzumab ozogamicin group versus 29.4% in the SC group [115]. Additionally, patients receiving INO achieved significantly higher rates of MRD negativity, longer PFS, and improved 2-year OS in an ad hoc analysis [114,115]. Furthermore, the analysis of pooled data from INO-VATE and the earlier phase I/II “Study1010” by Marks et al. revealed that salvage treatment with INO can provide a bridge to transplant in R/R B-ALL, and patients treated with INO were approximately one-third less likely to experience a relapse compared to those who received SC [116]. Hence, the role of INO as the bridge to HSCT is currently intensively investigated [117,118,119]. However, careful consideration is necessary when applying inotuzumab ozogamicin before the myeloablative conditioning regimen in HSCT recipients, due to unique adverse events, including multi-organ failure and a significant risk of veno-occlusive disease [120,121]. The encouraging data on the effectiveness of INO paved the way for studies investigating its use in first-line treatment alone or combined with reduced-intensity chemotherapy or other targeted therapies [122]. These clinical trials have predominantly targeted older adults, as they tend to experience poorer outcomes historically, often due to their limited tolerance to intensive chemotherapy, the frequent presence of high-risk disease characteristics, and reduced eligibility for HSCT [123]. The German Multicenter Study Group on Adult Acute Lymphoblastic Leukemia (GMALL) conducted the INITIAL-1 trial, an open-label phase II study, to evaluate the efficacy and safety of INO as an induction therapy for older adults with newly diagnosed Ph- B-ALL, the results of which were published recently [124]. Participants received up to three cycles of INO as induction therapy, followed by standard chemotherapy-based consolidation and maintenance treatments. The study demonstrated high remission rates, with all 43 patients achieving either CR or CRi, primarily after the first induction cycle, and MRD negativity improving to 74% by the third cycle. Survival outcomes were promising, with OS rates of 91% at one year and 81% at two years, and EFS at one year reaching 88%. The treatment was associated with common grade 3 or higher adverse events, including leukocytopenia, anemia, thrombocytopenia, and elevated liver enzymes. Only one case of suspected veno-occlusive disease occurred, highlighting a manageable safety profile for the therapy. These findings suggest that incorporating INO into induction regimens may improve outcomes for this patient population.

Polatuzumab vedotin (PV) is an mAb targeting CD79b delivering monomethyl auristatin E (MMAE), an anti-mitotic toxin, to cancer cells. In June 2019, the FDA granted accelerated approval for polatuzumab vedotin, in combination with bendamustine and rituximab, for treating adults with relapsed or refractory DLBCL who have undergone at least two prior therapies [125]. CD79b, a component of the B-cell receptor complex, is crucial for downstream signaling and is expressed in the majority of B-cell lymphomas, making it a promising immunotherapeutic target [126]. The approval of polatuzumab vedotin is a considerable advancement in treatment options for patients with R/R DLBCL, particularly for those ineligible for transplantation or CAR-T therapy [127]. Ongoing trials will determine whether bendamustine–rituximab chemoimmunotherapy is the optimal partner for polatuzumab in treating DLBCL patients. Early studies of various combinations have demonstrated antitumor activity and a manageable safety profile in R/R FL and frontline non-Hodgkin lymphoma settings [128,129]. Lately, we have observed a paradigm shift in the frontline treatment of DLBCL thanks to the POLLARIX clinical trial which challenged the current standard—the R-CHOP regimen [130]. In this trial, 879 newly diagnosed patients were randomly assigned to receive either the R-CHOP or PV-R-CHP regimen, in which vincristine was replaced by PV. After a median follow-up of 28.2 months, PFS was higher in the PV (76.7% compared to 70.2% in the R-CHOP group), although no significant differences in OS at the two-year mark were observed. Moreover, the safety profile of the novel PV regimen was similar to that in the R-CHOP arm. These results led to the FDA approving PV as a first-line treatment for adult patients who have previously untreated diffuse large B-cell lymphoma, not otherwise specified or high-grade B-cell lymphoma, and who have an International Prognostic Index score of 2 or greater, marking the first significant update to initial therapy for DLBCL in two decades [131].

Belantamab mafodotin (BelaMaf) was the only ADC approved for MM treatment, although other ADCs are under investigation. This ADC molecule is an mAb specific to BCMA and linked to the toxin auristatin F. Its use was intended for patients who have received at least four prior therapies, including an anti-CD38 mAb, a proteasome inhibitor, and an immunomodulatory agent [132]. The DREAMM-1 and DREAMM-2 studies demonstrated its effectiveness as a single agent in patients with advanced MM, achieving high responses (ORR of 32% and 35%) and long-term remission durations of approximately one year [133]. However, the phase III randomized DREAMM-3 trial, which aimed to evaluate the safety and efficacy of BelaMaf as a single agent compared to the PomDex (Pomalidomide and dexamethasone) regimen in patients with R/R MM, failed to show a PFS benefit [134]. These results were a base to withdraw BelaMaf by the FDA in November 2022 and subsequently, the conditional marketing authorization for Blenrep has not been renewed by the European Commission, as of October 2024. Nevertheless, the collected clinical data do not undermine completely the effectiveness of BelaMaf. Hence, many combination therapies are being tested to improve its safety and effectiveness profile. Notably, the latest results from the DREAMM-7 and DREAMM-8 studies are highly encouraging.

The DREAMM-7, phase III, multicenter, open-label, randomized study aimed to evaluate the efficacy and safety of BelaMaf in combination with bortezomib and dexamethasone (BorDex) compared to a combination of daratumumab and BorDex in patients with R/R MM. Results published this year indicate that the BelaMaf combination nearly tripled the median PFS compared to the standard daratumumab combination (36.6 months vs. 13.4 months), with a 59% reduction in the risk of disease progression or death [135]. These findings suggest that the BelaMaf plus BorDex regimen may offer a superior treatment option for R/R MM patients compared to the current standard of care.

The DREAMM-8 study, in turn, compared BelaMaf combined with pomalidomide and dexamethasone vs. pomalidomide plus bortezomib and dexamethasone (PVd) in R/R MM patients after one prior line of therapy. Similarly, the BelaMaf-based combination demonstrated superior performance compared to the control group in response rate, response durability, and PFS [136]. This combination significantly extended the time to disease progression or death compared to the PVd regimen. At a median follow-up of 21.8 months, the median PFS was not yet reached in the BelaMaf group, whereas it was 12.7 months in the PVd group. The ORR was higher in the BelaMaf arm, 77% compared to 72% in the PVd group. Notably, the CR was 40% in the BelaMaf group versus 16% in the PVd group. Among patients achieving CR or better, the MRD negativity rate was 23.9% in the BelaMaf group, compared to 4.8% in the PVd group, indicating deeper responses to the BelaMaf regimen. The safety and tolerability of this regimen were consistent with the known profiles of the individual agents. These results suggest that the BPd combination offers a significant improvement in progression-free survival and depth of response for patients with relapsed or refractory multiple myeloma, potentially redefining treatment standards in this setting. Additionally, a few other combination therapies are being tested to improve its effectiveness profile [137].

Loncastuximab tesirine consists of a CD19-targeting mAb linked to a pyrrolobenzodiazepine (PBD) dimer cytotoxic agent [138]. After binding to the CD19 antigen on B cells, the ADC is internalized, releasing the PBD dimer, which induces DNA cross-linking and prevents cancer cell replication, ultimately leading to cell death. The FDA approved loncastuximab tesirine in April 2021 for the treatment of R/R DLBCL, including DLBCL arising from low-grade lymphoma, in patients who have received at least two prior systemic therapies. In clinical trials, loncastuximab tesirine demonstrated significant efficacy in heavily pretreated patients, with durable responses, particularly in those with aggressive forms of DLBCL. A phase I study of loncastuximab tesirine in patients with R/R B-cell non-Hodgkin lymphoma (NHL) demonstrated an ORR of 45.6% in evaluable patients, with 26.7% achieving CR. Specifically, the ORR was 42.3% in patients with DLBCL, 46.7% in those with mantle cell lymphoma (MCL), and 78.6% in patients with FL [139].

Additionally, a multicenter, open-label, single-arm phase II trial (LOTIS-2) was conducted in patients with R/R DLBCL who had received two or more prior multiagent systemic treatments. The trial reported an ORR of 48.3%, a CR rate of 24.1%, and an OS of 9.9 months [140]. The results of LOTIS-1 and LOTIS-2 showed that loncastuximab tesirine had significant single-agent antitumor activity and durable response in R/R DLBCL patients, with acceptable safety and tolerability. Several clinical trials are ongoing to assess its safety and efficacy in NHL in various clinical settings, alone or in combination with other therapeutics [141].

### 2.4. Immune Checkpoint Inhibitors

Recognizing that neoplasm cells can hijack immune checkpoint pathways like cytotoxic T-lymphocyte-associated protein 4 (CTLA-4) and programmed death-1 (PD-1) to evade the immune system, immune checkpoint blockade therapy was developed as a novel immunotherapeutic approach [142]. Physiologically, immune checkpoints serve crucial functions in self-tolerance to avoid auto-immunity and tightly regulate the effector functions of activated T cells [143,144]. Immune checkpoint inhibitors (ICIs) are a class of mAbs that work by blocking immune checkpoint proteins from binding with partner molecules, thereby releasing immune breaks [145]. This strategy has proven effective against several solid tumors, including melanoma, non-small-cell lung cancer, renal cell carcinoma, and urothelial cancer, so far [146]. Over the past decade, various ICI drugs have received FDA approval including anti-CTLA-4 (ipilimumab), anti-PD-1 (pembrolizumab, nivolumab, and cemiplimab), and anti-PD-L1 (atezolizumab, avelumab, and durvalumab). However, their efficacy in HMs remains under extensive investigation. At present, the only FDA-approved ICI therapeutics are for classic Hodgkin lymphoma and primary mediastinal B-cell lymphoma. As knowledge of cancer biology and the tumor microenvironment (TME) expands, it is becoming evident that disease-specific factors, including the immune landscape of the disease, heavily shape both resistance and response to ICIs [147].

Classic Hodgkin lymphoma (cHL) has distinct biological features, marked by the presence of malignant Hodgkin and Reed–Sternberg (HRS) cells, which make up about 2% of the tumor cell population. The rest of its TME largely consists of reactive T cells and other immune cells, although these reactive immune cells exhibit an exhausted phenotype. This is caused mainly because of the over-expression of PDL-1 and PDL-2 on the malignant HRS cells, resulting in a loss of effector function of immune cells [148,149]. Hence, given its biology, the immune checkpoint blockade was first investigated in R/R cHL.

The first ICI tested in cHL was nivolumab, an anti-PD-1 mAb. The pivotal CheckMate-205 phase II trial reported an ORR of 69%, with 16% CR in 243 patients with R/R cHL following the failure of autoHSCT. The median time to response was approximately two months, and the median duration of response was 16.6 months [150]. The 5-year follow-up of the study demonstrated favorable OS and confirmed the efficacy and safety of nivolumab in R/R cHL after auto-HSCT failure [151]. Based on the results from the CheckMate-205 and CheckMate 039 trials, the FDA granted accelerated approval to nivolumab in 2016 for patients with R/R cHL who had failed autoHSCT and brentuximab vedotin treatment [152,153]. In addition, a breakthrough was achieved in the study comparing the combination regimen of nivolumab+AVD (doxorubicin, vinblastine, and dacarbazine) with current standard brentuximab+AVD in patients with advanced newly diagnosed Hodgkin’s lymphoma [154]. This multicenter, open-label, randomized trial enrolled 994 patients at least 12 years of age with stage III or IV previously untreated classic Hodgkin lymphoma. The outcomes of the study demonstrated significantly improved PFS in the nivolumab arm compared with the brentuximab vedotin arm (94% vs. 86% 1-year PFS rate). Additionally, the repeated analysis after one more year of follow-up revealed that the 2-year PFS rates were 92% in the nivolumab arm vs. 83% in the brentuximab vedotin arm. The 2-year EFS rate was 90% in the N+AVD arm and 81% in the BV+AVD arm. Although longer follow-up data are needed, this novel combination poses a promising option to revolutionize the treatment approach for elderly patients with advanced-stage cHL.

Pembrolizumab, another anti-PD-1 inhibitor, approved for use in melanoma, non-small-cell lung cancer, and head and neck cancers, has also been evaluated in HL treatment. In two clinical trials (KEYNOTE013 and KEYNOTE087), pembrolizumab has demonstrated significant efficacy in HL, with ORR ranging from 65% to 85%, depending on the cohort [155,156]. Furthermore, a phase III randomized controlled trial, Keynote-204, which compared pembrolizumab to brentuximab vedotin (BV) in the R/R setting, demonstrated an enhanced median PFS with pembrolizumab (13.2 months) compared to BV (8.3 months), with better tolerance to pembrolizumab vs. BV [157]. Based on these studies, the FDA granted accelerated approval to pembrolizumab for the treatment of cHL patients who are refractory or have relapsed after three or more prior therapies [158].

Additional studies with pembrolizumab are currently underway, focused on identifying the optimal combinations and timing for applying these agents in HL, and other lymphomas. Nevertheless, existing evidence highlights the remarkable responsiveness of HL to immune checkpoint blockade therapies [154,155,156,157,158]. The PD-1 blockade has also been investigated as a consolidation treatment following autoHSCT in high-risk patients with relapsed cHL, showing improved PFS, but more data are being awaited for optimal evaluation of these schemes [159,160,161].

Besides HL, PD-1 blockade has demonstrated significant efficacy in primary mediastinal and gray zone lymphomas, and Richter’s transformation of CLL [162,163,164,165,166]. Other lymphomas, particularly DLBCL in relapsed transplant-ineligible patients or as post-transplant consolidation, and FL, have shown limited benefit, as reviewed by others in [167,168]. ICIs have also provided minimal benefit in MM in studies performed so far, whether used alone or in combination with immunomodulatory agents like lenalidomide [169,170,171].

## 3. The Role of the Tumor Microenvironment in Regulating Responses to Antibody-Based Therapies in Hematological Malignancies

It is already well recognized that malignant cells form a dynamic network with surrounding components including different types of immune cells, stromal cells, blood and lymphatic vessels, secreted factors, and the extracellular matrix, all forming the tumor microenvironment (TME) [172]. Hence, in addition to its pharmacokinetic and pharmacodynamic properties, the TME plays a fundamental role in determining the efficacy of antibody-based therapeutics in hematological malignancies by influencing both direct tumor targeting and immune-mediated mechanisms [173]. A critical aspect is the TME’s immune cell composition, which varies substantially between different cancer types and affects disease outcomes and treatment responses [174,175]. Effector immune cells such as natural killer (NK) cells and macrophages are essential for antibody-dependent cellular cytotoxicity (ADCC) and phagocytosis (ADCP). However, their function can be hindered by immunosuppressive cells like regulatory T cells (Tregs) and myeloid-derived suppressor cells (MDSCs), which create a suppressive milieu that diminishes therapeutic efficacy [176]. Cytokine and chemokine networks within the TME further shape therapeutic responses. Pro-inflammatory cytokines can enhance immune activation, while anti-inflammatory mediators such as IL-10 or TGF-β can suppress it, thereby affecting the success of antibody therapies [177]. Particularly, the TME upregulates immune checkpoints, mainly PD-L1, which suppress T-cell activity, presenting a significant barrier to immune-based antibody therapies [178]. Other factors, including stromal barriers, extracellular matrix (ECM) components, metabolic challenges such as hypoxia, and antigen modulation, also contribute to therapeutic resistance. To counteract these challenges, combination strategies are being developed, such as pairing antibodies with immune checkpoint inhibitors, cytokine therapies, or agents targeting the stromal and immunosuppressive components of the TME [179]. By addressing the complex interplay between the TME and antibody therapies, these approaches aim to enhance treatment efficacy and overcome resistance mechanisms in hematological malignancies.

Physical components of the TME, including stromal barriers and extracellular matrix (ECM) components, can impede the penetration and distribution of therapeutic antibodies, reducing their effectiveness [180]. Metabolic challenges within the TME, such as hypoxia and nutrient depletion, can impair effector cell function, further diminishing the efficacy of antibody-dependent mechanisms [181]. To counteract these challenges, combination strategies are being developed. Pairing antibodies with immune checkpoint inhibitors aims to restore T-cell activity, while cytokine therapies seek to modulate the TME and enhance effector cell function. Additionally, agents targeting stromal components or ECM elements are being explored to improve antibody delivery and efficacy.

## 4. Conclusions and Future Directions

Cancer immunotherapy has transformed oncology care extending survival in historically fatal diseases. The number of patients eligible for immune-based therapies continues to grow, with immunotherapies now being used in first-line treatments [182]. Novel targets and combination therapies are on the way to expand cancer immunotherapy applications. Significant advancements in treatment and patient outcomes have been made across the full spectrum of HMs over the past two decades. Not only have cure rates for aggressive malignancies improved, but the most significant progress has been made in the management of typically incurable types of hematological cancers. Several HMs, such as CLL, MM, and certain types of non-Hodgkin lymphoma, are now chronic diseases that are treated with continuously administered therapies [183,184,185,186].

However, despite recent advances, immunotherapies do not benefit all patients. Resistance or lack of response are the most important drawbacks. Moreover, immunotherapies are still expensive and can cause serious immune-related side effects.

The advantages of antibody therapies arise from their high specificity, selectivity, and optimal binding affinity. The safety and effectiveness of these therapies are closely linked to the properties of their therapeutic targets, including their expression patterns and the functions they play in disease progression [187,188,189]. Although generally well tolerated, common toxicities observed in all mAbs trials are infusion-related reactions (IRRs), including anaphylactic reactions due to type-I hypersensitivity and side effects from the depletion of healthy cells expressing the same antigen. IRRs can range from mild symptoms such as fever, chills, muscle pain, and flushing to more severe and potentially life-threatening conditions like tachycardia or arrhythmias, difficulty breathing, low blood pressure, and shock [190,191]. Since most IRRs occur during the first infusion due to type-I hypersensitivity, slow infusion rates, and antiphlogistic pre-medication are optimal preventive measures during their application [192,193]. Nevertheless, depending on the severity of the IRR, most patients can be safely re-exposed to the mAb therapy.

From the mechanistic point of view, there is still a need for intensive preclinical research with respect to the subsequent clinical utility of mAbs and their biosimilars. For example, despite rituximab being widely used, considerable uncertainty remains about its mechanisms of action in vivo [194]. Hence, better preclinical models for assessment and the improvement of the pharmacokinetic and pharmacodynamic properties of mAbs are under investigation [195]. CAR T-cell therapies present an innovative approach that involves the ex vivo modification and activation of a patient’s T cells [196]. However, the specificity of CAR recognition and its ability to target tumor cells rely on mAb technology so the success of CAR T-cell therapies and most other immunotherapies relies on mAbs to directly or indirectly target specific antigens on cancer cells [15]. Hence, intensive research is pursued in designing the most effective mAb derivatives and fragments, with optimal size, half-life, valency, and affinity [7,197,198,199,200].

Advancements in protein engineering and production technologies have greatly enhanced the development of bispecific antibodies and their derivatives, positioning them as one of the fastest-growing categories of next-generation antibody therapeutics [196]. For a long time, the approval of bispecific T-cell engagers was far behind, with many molecules stuck in clinical trials. The last few years have brought some motion in this matter, opening the way for a few bispecific therapeutics to reach the clinic, targeting a range of TAAs and emerging in the treatment of multiple myeloma, non-Hodgkin’s lymphoma, acute myelogenous leukemia, and acute lymphoblastic leukemia [201,202,203]. The future development of BsAbs will focus on identifying and utilizing novel molecular targets, combinations, platforms, and geometric configurations. It will also involve integrating BsAbs with traditional biological drugs, other immunotherapies, and physical or chemical treatments [200,204,205]. However, despite their typically favorable safety profiles, certain toxicities like infections, cytokine release syndrome, myelosuppression, and neurotoxicity following BsAbs and T-cell engager therapy pose significant concerns, which should be further addressed in research [205].

Antibody–drug conjugates (ADCs) are a rapidly growing class of biotherapeutics that use antibodies to target and deliver cytotoxic drugs to tumor sites specifically. Despite the increasing interest in ADCs, challenges persist in improving their therapeutic index to enhance efficacy while reducing toxicity [206]. Recent advancements in manufacturing technology for antibody structure, payloads, linkers, innovative bioconjugation platforms, and cutting-edge analytical techniques are driving the future development of ADCs [207,208]. Moreover, continuous efforts are being made to identify the best combination therapies of ADCs with other agents [209].

The success of immune checkpoint inhibitors, anti-CTLA-4 in treating melanoma and anti-PD-1/PD-L1 in multiple types of cancer, has emphasized the potential of immunotherapy as a promising treatment option for patients with hematologic malignancies. mAbs targeting CTLA-4 or PD-1 have demonstrated notable clinical efficacy in patients with HMs, particularly in those with Hodgkin’s lymphoma and primary mediastinal B-cell lymphoma. These therapies are also being investigated for use in other hematological cancers and following allogeneic transplantation, although there is an anticipated risk of exacerbating graft-versus-host disease reactions. CTLA-4 and PD-1 blocking antibodies have demonstrated synergistic effectiveness, particularly in the treatment of solid tumors such as metastatic melanoma. Additionally, antibodies targeting other checkpoint molecules, including TIM3, TIGIT, and LAG-3, have been developed and are currently undergoing early clinical trials in patients with hematological malignancies.

Beyond these advancements, open research questions and areas for improvement remain in the field of mAb-based therapeutics. One promising area for future research is the development of therapies targeting novel antigens. Current HM immunotherapies have focused on a limited set of antigens, mainly CD19 and CD20, which are widely expressed on malignant cells. Expanding the range of targetable antigens, particularly those uniquely expressed on cancerous cells, could increase therapeutic specificity and minimize off-target effects. Identifying new tumor-associated antigens and understanding their expression patterns is crucial to broadening the therapeutic landscape, especially in the case of antigen escape cases or in treatment resistance [210,211,212]. Another key area of focus is improving the safety profile of bispecific antibodies. Although these therapies have demonstrated impressive clinical efficacy, they are frequently associated with adverse events, such as cytokine release syndrome (CRS) and neurotoxicity [213]. Addressing these safety concerns remains a high priority, and intensive research is conducted with respect of novel dosing regimens, engineering modifications, and co-treatment strategies to mitigate these side effects without compromising efficacy [214]. Optimizing the balance between immune activation and safety will play a crucial role in the widespread adoption of these therapies [90].

Immunotherapy is revolutionizing the treatment of certain hematological malignancies, providing cures even for patients with advanced stages of the disease. Current research is dedicated to improving both the safety and efficacy of these cutting-edge treatment modalities. Future developments in immunotherapy aim to boost its effectiveness, enhance safety, and broaden its use across a wider spectrum of hematological cancers.

## Figures and Tables

**Table 1:** Monoclonal antibodies approved in hemato-oncologic applications (October 2024).

Name	Brand Name	Targeted Antigen	Indication	Year Approved FDA	Year Approved EMA
rituximab	Rituxan	CD20	CLL, DLBCL, BL, BLL, B-ALL	1997	1998
ofatumumab	Arzerra	CD20	CLL	2009	2010 *
obinutuzumab	Gazyvaro/Gazyva	CD20	CLL, FL	2013	2014
elotuzumab	Empliciti	SLAMF7	R/R MM	2015	2016
daratumumab	Darzalex	CD38	MM	2016	2016
isatuximab	Sarclisa	CD38	MM	2020	2020
tafasitamab	Monjuvi	CD19	DLBCL	2020	2021

* withdrawn from the EU market. Abbreviations: CLL—chronic lymphocytic leukemia; DLBCL—diffuse large B-cell lymphoma; BL—Burkitt lymphoma; BLL—Burkitt-like lymphoma; B-ALL—B-cell acute lymphoblastic leukemia; FL—follicular lymphoma; MM—multiple myeloma, R/R—relapsed or refractory.

**Table 2:** Bispecific antibodies approved in hemato-oncologic applications (October 2024).

Name	Brand Name	Targeted Antigen	Indication	Year Approved FDA	Year Approved EMA
blinatumomab	Blincyto	CD3xCD19	B-ALL Ph^−^	2014	2015
mosunetuzumab	Lunsumio	CD3xCD20	R/R FL	2022	2022
teclistamab	Tecvayli	CD3xBCMA	R/R MM	2022	2022
epcoritamab	Epkinly	CD3xCD20	R/R DLBCL	2023	2023
glofitamab	Columvi	CD3xCD20	R/R DLBCL, LBL	2023	2023
elranatamab	Elrexfio	CD3xBCMA	R/R MM	2023	2023
talquetamab	Talvey	CD3xGPRC5D	R/R MM	2023	2023

Abbreviations: B-ALL—B-cell acute lymphoblastic leukemia; FL—follicular lymphoma; MM—multiple myeloma; DLBCL—diffuse large B-cell lymphoma; LBL—large B-cell lymphoma; R/R—relapsed or refractory; Ph^−^—Philadelphia chromosome-negative.

**Table 3:** Antibody–drug conjugates approved in hemato-oncologic applications (October 2024).

Name	Brand Name	Targeted Antigen	Payload	Indication	Year Approved FDA	Year Approved EMA
gemtuzumab ozogamicin	Mylotarg	CD33	calicheamicin	AML	2000, 2017	2018
brentuximab vedotin	Adcetris	CD30	monomethyl auristatin E	HL	2011	2012
inotuzumab ozogamicin	Besponsa	CD22	calicheamicin	R/R CD22+ B-ALL	2017	2017
polatuzumab vedotin	Polivy	CD79b	monomethyl auristatin E	DLBCL	2019	2020
belantamab mafodotin	Blenrep	BCMA	monomethyl auristatin F	R/R MM	2020 *	2020 *
loncastuximab tesirine	Zynlonta	CD19	pyrrolobenzodiazepine dimer SG3199	R/R DLBCL, R/R HGBL	2021	2022

* withdrawn from the market. Abbreviations: AML—acute myeloid leukemia; HL—Hodkin lymphoma; B-ALL—B-cell acute lymphoblastic leukemia; DLBCL—diffuse large B-cell lymphoma; MM—multiple myeoloma; HGBL—high-grade B-cell lymphoma; R/R—relapsed or refractory.

## References

[1] Couzin-Frankel J. (2013). Cancer Immunotherapy. Science.

[2] Huang P.-W., Chang J.W.-C. (2019). Immune Checkpoint Inhibitors Win the 2018 Nobel Prize. Biomed. J..

[3] Coley W.B. (1991). The Treatment of Malignant Tumors by Repeated Inoculations of Erysipelas. With a Report of Ten Original Cases. 1893. Clin. Orthop. Relat. Res..

[4] Dunn G.P., Old L.J., Schreiber R.D. (2004). The Three Es of Cancer Immunoediting. Annu. Rev. Immunol..

[5] Weiden P.L., Flournoy N., Thomas E.D., Prentice R., Fefer A., Buckner C.D., Storb R. (1979). Antileukemic Effect of Graft-versus-Host Disease in Human Recipients of Allogeneic-Marrow Grafts. N. Engl. J. Med..

[6] Maurer K., Antin J.H. (2024). The Graft versus Leukemia Effect: Donor Lymphocyte Infusions and Cellular Therapy. Front. Immunol..

[7] Strohl W.R. (2018). Current Progress in Innovative Engineered Antibodies. Protein Cell.

[8] Sifniotis V., Cruz E., Eroglu B., Kayser V. (2019). Current Advancements in Addressing Key Challenges of Therapeutic Antibody Design, Manufacture, and Formulation. Antibodies.

[9] Kaplon H., Crescioli S., Chenoweth A., Visweswaraiah J., Reichert J.M. (2023). Antibodies to Watch in 2023. MAbs.

[10] Crescioli S., Kaplon H., Chenoweth A., Wang L., Visweswaraiah J., Reichert J.M. (2024). Antibodies to Watch in 2024. MAbs.

[11] Grilo A.L., Mantalaris A. (2019). The Increasingly Human and Profitable Monoclonal Antibody Market. Trends Biotechnol..

[12] Kinch M.S., Kraft Z., Schwartz T. (2023). Monoclonal Antibodies: Trends in Therapeutic Success and Commercial Focus. Drug Discov. Today.

[13] Köhler G., Milstein C. (1975). Continuous Cultures of Fused Cells Secreting Antibody of Predefined Specificity. Nature.

[14] Harding F.A., Stickler M.M., Razo J., DuBridge R.B. (2010). The Immunogenicity of Humanized and Fully Human Antibodies: Residual Immunogenicity Resides in the CDR Regions. MAbs.

[15] Weiner G.J. (2015). Building Better Monoclonal Antibody-Based Therapeutics. Nat. Rev. Cancer.

[16] Almagro J.C., Daniels-Wells T.R., Perez-Tapia S.M., Penichet M.L. (2018). Progress and Challenges in the Design and Clinical Development of Antibodies for Cancer Therapy. Front. Immunol..

[17] Almagro J.C., Pedraza-Escalona M., Arrieta H.I., Pérez-Tapia S.M. (2019). Phage Display Libraries for Antibody Therapeutic Discovery and Development. Antibodies.

[18] Najfeld V. (2018). Conventional and Molecular Cytogenomic Basis of Hematologic Malignancies. Hematology: Basic Principles and Practice.

[19] Hartmann L., Metzeler K.H. (2019). Clonal Hematopoiesis and Preleukemia-Genetics, Biology, and Clinical Implications. Genes Chromosomes Cancer.

[20] Niroula A., Sekar A., Murakami M.A., Trinder M., Agrawal M., Wong W.J., Bick A.G., Uddin M.M., Gibson C.J., Griffin G.K. (2021). Distinction of Lymphoid and Myeloid Clonal Hematopoiesis. Nat. Med..

[21] Filipek-Gorzała J., Kwiecińska P., Szade A., Szade K. (2024). The Dark Side of Stemness—The Role of Hematopoietic Stem Cells in Development of Blood Malignancies. Front. Oncol..

[22] Hu D., Shilatifard A. (2016). Epigenetics of Hematopoiesis and Hematological Malignancies. Genes Dev..

[23] Taylor J., Xiao W., Abdel-Wahab O. (2017). Diagnosis and Classification of Hematologic Malignancies on the Basis of Genetics. Blood.

[24] Jaffe E.S., Harris N.L., Stein H., Vardiman J.W. (2001). Pathology and Genetics of Tumours of Haematopoietic and Lymphoid Tissues.

[25] Salama M.E., Hoffman R. (2017). Progress in the Classification of Hematopoietic and Lymphoid Neoplasms: Clinical Implications. Hematology: Basic Principles and Practice.

[26] Bray F., Laversanne M., Sung H., Ferlay J., Siegel R.L., Soerjomataram I., Jemal A. (2024). Global Cancer Statistics 2022: GLOBOCAN Estimates of Incidence and Mortality Worldwide for 36 Cancers in 185 Countries. CA. Cancer J. Clin..

[27] Pulte D., Jansen L., Brenner H. (2020). Changes in Long Term Survival after Diagnosis with Common Hematologic Malignancies in the Early 21st Century. Blood Cancer J..

[28] Maloney D.G., Grillo-López A.J., White C.A., Bodkin D., Schilder R.J., Neidhart J.A., Janakiraman N., Foon K.A., Liles T.-M., Dallaire B.K. (1997). IDEC-C2B8 (Rituximab) Anti-CD20 Monoclonal Antibody Therapy in Patients With Relapsed Low-Grade Non-Hodgkin’s Lymphoma. Blood.

[29] Weiner G.J. (2010). Rituximab: Mechanism of Action. Semin. Hematol..

[30] Nadler L.M., Ritz J., Hardy R., Pesando J.M., Schlossman S.F., Stashenko P. (1981). A Unique Cell Surface Antigen Identifying Lymphoid Malignancies of B Cell Origin. J. Clin. Investig..

[31] Johnson P., Glennie M. (2003). The Mechanisms of Action of Rituximab in the Elimination of Tumor Cells. Semin. Oncol..

[32] Chen D.R., Cohen P.L. (2012). Living Life without B Cells: Is Repeated B-Cell Depletion a Safe and Effective Long-Term Treatment Plan for Rheumatoid Arthritis?. Int. J. Clin. Rheumtol..

[33] Athni T.S., Barmettler S. (2023). Hypogammaglobulinemia, Late-Onset Neutropenia, and Infections Following Rituximab. Ann. Allergy. Asthma Immunol..

[34] Salles G., Barrett M., Foà R., Maurer J., O’Brien S., Valente N., Wenger M., Maloney D.G. (2017). Rituximab in B-Cell Hematologic Malignancies: A Review of 20 Years of Clinical Experience. Adv. Ther..

[35] Pierpont T.M., Limper C.B., Richards K.L. (2018). Past, Present, and Future of Rituximab-The World’s First Oncology Monoclonal Antibody Therapy. Front. Oncol..

[36] Al Hamed R., Bazarbachi A.H., Mohty M. (2020). Epstein-Barr Virus-Related Post-Transplant Lymphoproliferative Disease (EBV-PTLD) in the Setting of Allogeneic Stem Cell Transplantation: A Comprehensive Review from Pathogenesis to Forthcoming Treatment Modalities. Bone Marrow Transplant..

[37] Marjańska A., Pogorzała M., Dziedzic M., Czyżewski K., Richert-Przygońska M., Dębski R., Bogiel T. (2024). Impact of prophylaxis with rituximab on EBV-related complications after allogeneic hematopoietic cell transplantation in children. Front. Immunol..

[38] Wierda W.G., Kipps T.J., Mayer J., Stilgenbauer S., Williams C.D., Hellmann A., Robak T., Furman R.R., Hillmen P., Trneny M. (2010). Ofatumumab as Single-Agent CD20 Immunotherapy in Fludarabine-Refractory Chronic Lymphocytic Leukemia. J. Clin. Oncol..

[39] Hillmen P., Robak T., Janssens A., Babu K.G., Kloczko J., Grosicki S., Doubek M., Panagiotidis P., Kimby E., Schuh A. (2015). Chlorambucil plus Ofatumumab versus Chlorambucil Alone in Previously Untreated Patients with Chronic Lymphocytic Leukaemia (COMPLEMENT 1): A Randomised, Multicentre, Open-Label Phase 3 Trial. Lancet.

[40] Kang C., Blair H.A. (2022). Ofatumumab: A Review in Relapsing Forms of Multiple Sclerosis. Drugs.

[41] Goede V., Fischer K., Busch R., Engelke A., Eichhorst B., Wendtner C.M., Chagorova T., de la Serna J., Dilhuydy M.-S., Illmer T. (2014). Obinutuzumab plus Chlorambucil in Patients with CLL and Coexisting Conditions. N. Engl. J. Med..

[42] Sehn L.H., Chua N., Mayer J., Dueck G., Trněný M., Bouabdallah K., Fowler N., Delwail V., Press O., Salles G. (2016). Obinutuzumab plus Bendamustine versus Bendamustine Monotherapy in Patients with Rituximab-Refractory Indolent Non-Hodgkin Lymphoma (GADOLIN): A Randomised, Controlled, Open-Label, Multicentre, Phase 3 Trial. Lancet. Oncol..

[43] Mössner E., Brünker P., Moser S., Püntener U., Schmidt C., Herter S., Grau R., Gerdes C., Nopora A., Van Puijenbroek E. (2010). Increasing the Efficacy of CD20 Antibody Therapy through the Engineering of a New Type II Anti-CD20 Antibody with Enhanced Direct and Immune Effector Cell–Mediated B-Cell Cytotoxicity. Blood.

[44] Townsend W., Hiddemann W., Buske C., Cartron G., Cunningham D., Dyer M.J.S., Gribben J.G., Phillips E.H., Dreyling M., Seymour J.F. (2023). Obinutuzumab Versus Rituximab Immunochemotherapy in Previously Untreated INHL: Final Results From the GALLIUM Study. HemaSphere.

[45] Sehn L.H., Martelli M., Trněný M., Liu W., Bolen C.R., Knapp A., Sahin D., Sellam G., Vitolo U., Sehn L.H. (2020). A Randomized, Open-Label, Phase III Study of Obinutuzumab or Rituximab plus CHOP in Patients with Previously Untreated Diffuse Large B-Cell Lymphoma: Final Analysis of GOYA. J. Hematol. Oncol..

[46] Le Gouill S., Ghesquières H., Oberic L., Morschhauser F., Tilly H., Ribrag V., Lamy T., Thieblemont C., Maisonneuve H., Gressin R. (2021). Obinutuzumab vs Rituximab for Advanced DLBCL: A PET-Guided and Randomized Phase 3 Study by LYSA. Blood.

[47] Davies A., Kater A.P., Sharman J.P., Stilgenbauer S., Vitolo U., Klein C., Parreira J., Salles G. (2022). Obinutuzumab in the Treatment of B-Cell Malignancies: A Comprehensive Review. Futur. Oncol..

[48] Sarkozy C., Callanan M., Thieblemont C., Obéric L., Burroni B., Bouabdallah K., Damaj G., Tessoulin B., Ribrag V., Houot R. (2024). Obinutuzumab vs Rituximab for Transplant-Eligible Patients with Mantle Cell Lymphoma. Blood.

[49] Jurczak W., Długosz Danecka M., Buske C. (2019). Rituximab Biosimilars for Lymphoma in Europe. Expert Opin. Biol. Ther..

[50] Olejarz W., Basak G. (2023). Emerging Therapeutic Targets and Drug Resistance Mechanisms in Immunotherapy of Hematological Malignancies. Cancers.

[51] Lonial S., Dimopoulos M., Palumbo A., White D., Grosicki S., Spicka I., Walter-Croneck A., Moreau P., Mateos M.-V., Magen H. (2015). Elotuzumab Therapy for Relapsed or Refractory Multiple Myeloma. N. Engl. J. Med..

[52] Chen W.C., Kanate A.S., Craig M., Petros W.P., Hazlehurst L.A. (2017). Emerging Combination Therapies for the Management of Multiple Myeloma: The Role of Elotuzumab. Cancer Manag. Res..

[53] Costa F., Palma B.D., Giuliani N. (2019). CD38 Expression by Myeloma Cells and Its Role in the Context of Bone Marrow Microenvironment: Modulation by Therapeutic Agents. Cells.

[54] McKeage K. (2016). Daratumumab: First Global Approval. Drugs.

[55] Dhillon S. (2020). Isatuximab: First Approval. Drugs.

[56] Frampton J.E. (2021). Isatuximab: A Review of Its Use in Multiple Myeloma. Target. Oncol..

[57] Facon T., Dimopoulos M.-A., Leleu X.P., Beksac M., Pour L., Hájek R., Liu Z., Minarik J., Moreau P., Romejko-Jarosinska J. (2024). Isatuximab, Bortezomib, Lenalidomide, and Dexamethasone for Multiple Myeloma. N. Engl. J. Med..

[58] Sarkozy C., Sehn L.H. (2019). New Drugs for the Management of Relapsed or Refractory Diffuse Large B-Cell Lymphoma. Ann. Lymphoma.

[59] Hoy S.M. (2020). Tafasitamab: First Approval. Drugs.

[60] Salles G., Duell J., González Barca E., Tournilhac O., Jurczak W., Liberati A.M., Nagy Z., Obr A., Gaidano G., André M. (2020). Tafasitamab plus Lenalidomide in Relapsed or Refractory Diffuse Large B-Cell Lymphoma (L-MIND): A Multicentre, Prospective, Single-Arm, Phase 2 Study. Lancet. Oncol..

[61] Duell J., Maddocks K.J., González-Barca E., Jurczak W., Liberati A.M., de Vos S., Nagy Z., Obr A., Gaidano G., Abrisqueta P. (2021). Long-Term L-MIND Study Outcomes of Tafasitamab from the(MOR208) Phase II plus Lenalidomide in Patients with Relapsed or Refractory Diffuse Large B-Cell Lymphoma. Haematologica.

[62] Salles G., Długosz-Danecka M., Ghesquières H., Jurczak W. (2021). Tafasitamab for the Treatment of Relapsed or Refractory Diffuse Large B-Cell Lymphoma. Expert Opin. Biol. Ther..

[63] Duell J., Westin J. (2025). The future of immunotherapy for diffuse large B-cell lymphoma. Int. J. Cancer.

[64] Chames P., Baty D. (2009). Bispecific Antibodies for Cancer Therapy: The Light at the End of the Tunnel?. MAbs.

[65] Del Bano J., Chames P., Baty D., Kerfelec B. (2015). Taking up Cancer Immunotherapy Challenges: Bispecific Antibodies, the Path Forward?. Antibodies.

[66] Yang F., Wen W., Qin W. (2017). Bispecific Antibodies as a Development Platform for New Concepts and Treatment Strategies. Int. J. Mol. Sci..

[67] Velasquez M.P., Bonifant C.L., Gottschalk S. (2018). Redirecting T Cells to Hematological Malignancies with Bispecific Antibodies. Blood.

[68] Cech P., Skórka K., Dziki L., Giannopoulos K. (2024). T-Cell Engagers—The Structure and Functional Principle and Application in Hematological Malignancies. Cancers.

[69] Labrijn A.F., Janmaat M.L., Reichert J.M., Parren P.W.H.I. (2019). Bispecific Antibodies: A Mechanistic Review of the Pipeline. Nat. Rev. Drug Discov..

[70] Thakur A., Huang M., Lum L.G. (2018). Bispecific Antibody Based Therapeutics: Strengths and Challenges. Blood Rev..

[71] Stamova S., Koristka S., Keil J., Arndt C., Feldmann A., Michalk I., Bartsch H., Bippes C.C., Schmitz M., Cartellieri M. (2012). Cancer Immunotherapy by Retargeting of Immune Effector Cells via Recombinant Bispecific Antibody Constructs. Antibodies.

[72] Blanco B., Domínguez-Alonso C., Alvarez-Vallina L. (2021). Bispecific Immunomodulatory Antibodies for Cancer Immunotherapy. Clin. Cancer Res..

[73] Huehls A.M., Coupet T.A., Sentman C.L. (2015). Bispecific T-Cell Engagers for Cancer Immunotherapy. Immunol. Cell Biol..

[74] Przepiorka D., Ko C.W., Deisseroth A., Yancey C.L., Candau-Chacon R., Chiu H.J., Gehrke B.J., Gomez-Broughton C., Kane R.C., Kirshner S. (2015). FDA Approval: Blinatumomab. Clin. Cancer Res..

[75] Portell C.A., Wenzell C.M., Advani A.S. (2013). Clinical and Pharmacologic Aspects of Blinatumomab in the Treatment of B-Cell Acute Lymphoblastic Leukemia. Clin. Pharmacol. Adv. Appl..

[76] Carter R.H., Wang Y., Brooks S. (2002). Role of CD19 Signal Transduction in B Cell Biology. Immunol. Res..

[77] Otero D.C., Anzelon A.N., Rickert R.C. (2003). CD19 Function in Early and Late B Cell Development: I. Maintenance of Follicular and Marginal Zone B Cells Requires CD19-Dependent Survival Signals. J. Immunol..

[78] Eibel H., Kraus H., Sic H., Kienzler A.K., Rizzi M. (2014). B Cell Biology: An Overview Topical Collection on Basic and Applied Science. Curr. Allergy Asthma Rep..

[79] Hammer O. (2012). CD19 as an Attractive Target for Antibody-Based Therapy. MAbs.

[80] Ali S., Moreau A., Melchiorri D., Camarero J., Josephson F., Olimpier O., Bergh J., Karres D., Tzogani K., Gisselbrecht C. (2020). Blinatumomab for Acute Lymphoblastic Leukemia: The First Bispecific T-Cell Engager Antibody to Be Approved by the EMA for Minimal Residual Disease. Oncologist.

[81] Boissel N., Chiaretti S., Papayannidis C., Ribera J.M., Bassan R., Sokolov A.N., Alam N., Brescianini A., Pezzani I., Kreuzbauer G. (2023). Real-World Use of Blinatumomab in Adult Patients with B-Cell Acute Lymphoblastic Leukemia in Clinical Practice: Results from the NEUF Study. Blood Cancer J..

[82] Halford Z., Coalter C., Gresham V., Brown T. (2021). A Systematic Review of Blinatumomab in the Treatment of Acute Lymphoblastic Leukemia: Engaging an Old Problem With New Solutions. Ann. Pharmacother..

[83] van der Sluis I.M., de Lorenzo P., Kotecha R.S., Attarbaschi A., Escherich G., Nysom K., Stary J., Ferster A., Brethon B., Locatelli F. (2023). Blinatumomab Added to Chemotherapy in Infant Lymphoblastic Leukemia. N. Engl. J. Med..

[84] Sayyed A., Chen C., Gerbitz A., Kim D.D.H., Kumar R., Lam W., Law A.D., Lipton J.H., Michelis F.V., Novitzky-Basso I. (2024). Pretransplant Blinatumomab Improves Outcomes in B Cell Acute Lymphoblastic Leukemia Patients Who Undergo Allogeneic Hematopoietic Cell Transplantation. Transplant. Cell. Ther..

[85] Llaurador G., Shaver K., Wu M., Wang T., Gillispie A., Doherty E., Craddock J., Read J., Yassine K., Morales E. (2024). Blinatumomab Therapy Is Associated with Favorable Outcomes after Allogeneic Hematopoietic Cell Transplantation in Pediatric Patients with B Cell Acute Lymphoblastic Leukemia. Transplant. Cell. Ther..

[86] Locatelli F., Zugmaier G., Rizzari C., Morris J.D., Gruhn B., Klingebiel T., Parasole R., Linderkamp C., Flotho C., Petit A. (2021). Effect of Blinatumomab vs Chemotherapy on Event-Free Survival Among Children With High-Risk First-Relapse B-Cell Acute Lymphoblastic Leukemia: A Randomized Clinical Trial. JAMA.

[87] Locatelli F., Zugmaier G., Rizzari C., Morris J.D., Gruhn B., Klingebiel T., Parasole R., Linderkamp C., Flotho C., Petit A. (2023). Improved Survival and MRD Remission with Blinatumomab vs. Chemotherapy in Children with First High-Risk Relapse B-ALL. Leukemia.

[88] Peters C., Bruno A., Rizzari C., Brescianini A., Von Stackelberg A., Linderkamp C., Zeng Y., Zugmaier G., Locatelli F. (2024). Blinatumomab Is Associated with Better Post-Transplant Outcome than Chemotherapy in Children with High-Risk First-Relapse B-Cell Acute Lymphoblastic Leukemia Irrespective of the Conditioning Regimen. Haematologica.

[89] Peters C., Dalle J.H., Locatelli F., Poetschger U., Sedlacek P., Buechner J., Shaw P.J., Staciuk R., Ifversen M., Pichler H. (2021). Total Body Irradiation or Chemotherapy Conditioning in Childhood ALL: A Multinational, Randomized, Noninferiority Phase III Study. J. Clin. Oncol..

[90] Abou Dalle I., Dulery R., Moukalled N., Ricard L., Stocker N., El-Cheikh J., Mohty M., Bazarbachi A. (2024). Bi- and Tri-Specific Antibodies in Non-Hodgkin Lymphoma: Current Data and Perspectives. Blood Cancer J..

[91] Carpenter R.O., Evbuomwan M.O., Pittaluga S., Rose J.J., Raffeld M., Yang S., Gress R.E., Hakim F.T., Kochenderfer J.N. (2013). B-Cell Maturation Antigen Is a Promising Target for Adoptive T-Cell Therapy of Multiple Myeloma. Clin. Cancer Res..

[92] Abramson H.N. (2020). B-Cell Maturation Antigen (BCMA) as a Target for New Drug Development in Relapsed and/or Refractory Multiple Myeloma. Int. J. Mol. Sci..

[93] Kang C. (2022). Teclistamab: First Approval. Drugs.

[94] Moreau P., Garfall A.L., van de Donk N.W.C.J., Nahi H., San-Miguel J.F., Oriol A., Nooka A.K., Martin T., Rosinol L., Chari A. (2022). Teclistamab in Relapsed or Refractory Multiple Myeloma. N. Engl. J. Med..

[95] Dhillon S. (2023). Elranatamab: First Approval. Drugs.

[96] Bahlis N.J., Costello C.L., Raje N.S., Levy M.Y., Dholaria B., Solh M., Tomasson M.H., Damore M.A., Jiang S., Basu C. (2023). Elranatamab in Relapsed or Refractory Multiple Myeloma: The MagnetisMM-1 Phase 1 Trial. Nat. Med..

[97] Lesokhin A.M., Tomasson M.H., Arnulf B., Bahlis N.J., Miles Prince H., Niesvizky R., Rodrίguez-Otero P., Martinez-Lopez J., Koehne G., Touzeau C. (2023). Elranatamab in Relapsed or Refractory Multiple Myeloma: Phase 2 MagnetisMM-3 Trial Results. Nat. Med..

[98] Tomasson M., Iida S., Niesvizky R., Mohty M., Bahlis N.J., Martinez-Lopez J., Koehne G., Rodriguez Otero P., Prince H.M., Viqueira A. (2023). Long-Term Efficacy and Safety of Elranatamab Monotherapy in the Phase 2 Magnetismm-3 Trial in Relapsed or Refractory Multiple Myeloma (RRMM). Blood.

[99] van de Donk N.W.C.J., O’Neill C., de Ruijter M.E.M., Verkleij C.P.M., Zweegman S. (2023). T-Cell Redirecting Bispecific and Trispecific Antibodies in Multiple Myeloma beyond BCMA. Curr. Opin. Oncol..

[100] Chari A., Touzeau C., Schinke C., Minnema M.C., Berdeja J., Oriol A., Van De Donk N.W., Rodriguez Otero P., Askari E., Mateos M.-V. (2022). Talquetamab, a G Protein-Coupled Receptor Family C Group 5 Member D x CD3 Bispecific Antibody, in Patients with Relapsed/Refractory Multiple Myeloma (RRMM): Phase 1/2 Results from MonumenTAL-1. Blood.

[101] Jakubowiak A.J., Anguille S., Karlin L., Chari A., Schinke C., Rasche L., San-Miguel J., Campagna M., Hilder B.W., Masterson T.J. (2023). Updated Results of Talquetamab, a GPRC5D×CD3 Bispecific Antibody, in Patients with Relapsed/Refractory Multiple Myeloma with Prior Exposure to T-Cell Redirecting Therapies: Results of the Phase 1/2 MonumenTAL-1 Study. Blood.

[102] Sanchez L., Schinke C., Krishnan A., Berdeja J.G., van de Donk N.W., Mateos M.V., Chari A., Parekh S., Mouhieddine T.H., Jagannath S. (2023). Clinical Outcomes of Subsequent Therapies in Patients with Relapsed/Refractory Multiple Myeloma Following Talquetamab Treatment: Analyses from the Phase 1/2 MonumenTAL-1 Study. Blood.

[103] Beck A., Goetsch L., Dumontet C., Corvaïa N. (2017). Strategies and Challenges for the next Generation of Antibody-Drug Conjugates. Nat. Rev. Drug Discov..

[104] Larson S.M., Carrasquillo J.A., Cheung N.K.V., Press O.W. (2015). Radioimmunotherapy of Human Tumours. Nat. Rev. Cancer.

[105] Baron J., Wang E.S. (2018). Gemtuzumab Ozogamicin for the Treatment of Acute Myeloid Leukemia. Expert Rev. Clin. Pharmacol..

[106] Fostvedt L.K., Hibma J.E., Masters J.C., Vandendries E., Ruiz-Garcia A. (2019). Pharmacokinetic/Pharmacodynamic Modeling to Support the Re-approval of Gemtuzumab Ozogamicin. Clin. Pharmacol. Ther..

[107] Nguyen T.D., Bordeau B.M., Balthasar J.P. (2023). Mechanisms of ADC Toxicity and Strategies to Increase ADC Tolerability. Cancers.

[108] Birrer M.J., Moore K.N., Betella I., Bates R.C. (2019). Antibody-Drug Conjugate-Based Therapeutics: State of the Science. J. Natl. Cancer Inst..

[109] Chau C.H., Steeg P.S., Figg W.D. (2019). Antibody-Drug Conjugates for Cancer. Lancet.

[110] De Claro R.A., McGinn K., Kwitkowski V., Bullock J., Khandelwal A., Habtemariam B., Ouyang Y., Saber H., Lee K., Koti K. (2012). U.S. Food and Drug Administration Approval Summary: Brentuximab Vedotin for the Treatment of Relapsed Hodgkin Lymphoma or Relapsed Systemic Anaplastic Large-Cell Lymphoma. Clin. Cancer Res..

[111] Connors J.M., Jurczak W., Straus D.J., Ansell S.M., Kim W.S., Gallamini A., Younes A., Alekseev S., Illés Á., Picardi M. (2018). Brentuximab Vedotin with Chemotherapy for Stage III or IV Hodgkin’s Lymphoma. N. Engl. J. Med..

[112] Straus D.J., Długosz-Danecka M., Connors J.M., Alekseev S., Illés Á., Picardi M., Lech-Maranda E., Feldman T., Smolewski P., Savage K.J. (2021). Brentuximab Vedotin with Chemotherapy for Stage III or IV Classical Hodgkin Lymphoma (ECHELON-1): 5-Year Update of an International, Open-Label, Randomised, Phase 3 Trial. Lancet Haematol..

[113] Borchmann P., Ferdinandus J., Schneider G., Moccia A., Greil R., Hertzberg M., Schaub V., Hüttmann A., Keil F., Dierlamm J. (2024). Assessing the Efficacy and Tolerability of PET-Guided BrECADD versus EBEACOPP in Advanced-Stage, Classical Hodgkin Lymphoma (HD21): A Randomised, Multicentre, Parallel, Open-Label, Phase 3 Trial. Lancet.

[114] Lamb Y.N. (2017). Inotuzumab Ozogamicin: First Global Approval. Drugs.

[115] Kantarjian H.M., DeAngelo D.J., Stelljes M., Liedtke M., Stock W., Gökbuget N., O’Brien S.M., Jabbour E., Wang T., Liang White J. (2019). Inotuzumab Ozogamicin versus Standard of Care in Relapsed or Refractory Acute Lymphoblastic Leukemia: Final Report and Long-Term Survival Follow-up from the Randomized, Phase 3 INO-VATE Study. Cancer.

[116] Marks D.I., Kebriaei P., Stelljes M., Gökbuget N., Kantarjian H., Advani A.S., Merchant A., Stock W., Cassaday R.D., Wang T. (2019). Outcomes of Allogeneic Stem Cell Transplantation after Inotuzumab Ozogamicin Treatment for Relapsed or Refractory Acute Lymphoblastic Leukemia. Biol. Blood Marrow Transplant..

[117] Stelmach P., Wethmar K., Groth C., Wenge D.V., Albring J., Mikesch J.H., Schliemann C., Reicherts C., Berdel W.E., Lenz G. (2020). Blinatumomab or Inotuzumab Ozogamicin as Bridge to Allogeneic Stem Cell Transplantation for Relapsed or Refractory B-Lineage Acute Lymphoblastic Leukemia: A Retrospective Single-Center Analysis. Clin. Lymphoma. Myeloma Leuk..

[118] Curran E., O’Brien M. (2020). Role of Blinatumomab, Inotuzumab, and CAR T-Cells: Which to Choose and How to Sequence for Patients with Relapsed Disease. Semin. Hematol..

[119] Wudhikarn K., King A.C., Geyer M.B., Roshal M., Bernal Y., Gyurkocza B., Perales M.A., Park J.H. (2022). Outcomes of Relapsed B-Cell Acute Lymphoblastic Leukemia after Sequential Treatment with Blinatumomab and Inotuzumab. Blood Adv..

[120] Kebriaei P., Cutler C., De Lima M., Giralt S., Lee S.J., Marks D., Merchant A., Stock W., Van Besien K., Stelljes M. (2018). Management of Important Adverse Events Associated with Inotuzumab Ozogamicin: Expert Panel Review. Bone Marrow Transplant..

[121] Kayser S., Sartor C., Giglio F., Bruno A., Webster J., Chiusolo P., Saraceni F., Guerzoni S., Pochintesta L., Borlenghi E. (2024). Impact of Inotuzumab Ozogamicin on Outcome in Relapsed or Refractory Acute B-Cell Lymphoblastic Leukemia Patients Prior to Allogeneic Hematopoietic Stem Cell Transplantation and Risk of Sinusoidal Obstruction Syndrome/Venous Occlusive Disease. Haematologica.

[122] Kantarjian H.M., Boissel N., Papayannidis C., Luskin M.R., Stelljes M., Advani A.S., Jabbour E.J., Ribera J.M., Marks D.I. (2024). Inotuzumab Ozogamicin in Adult Acute Lymphoblastic Leukemia: Development, Current Status, and Future Directions. Cancer.

[123] Wenge D.V., Wethmar K., Klar C.A., Kolve H., Sauer T., Angenendt L., Evers G., Call S., Kerkhoff A., Khandanpour C. (2022). Characteristics and Outcome of Elderly Patients (>55 Years) with Acute Lymphoblastic Leukemia. Cancers.

[124] Stelljes M., Raffel S., Alakel N., Wäsch R., Kondakci M., Scholl S., Rank A., Hänel M., Spriewald B., Hanoun M. (2024). Inotuzumab Ozogamicin as Induction Therapy for Patients Older than 55 Years with Philadelphia Chromosome–Negative B-Precursor ALL. J. Clin. Oncol..

[125] Deeks E.D. (2019). Polatuzumab Vedotin: First Global Approval. Drugs.

[126] Pfeifer M., Zheng B., Erdmann T., Koeppen H., Mccord R., Grau M., Staiger A., Chai A., Sandmann T., Madle H. (2015). Anti-CD22 and Anti-CD79B Antibody Drug Conjugates Are Active in Different Molecular Diffuse Large B-Cell Lymphoma Subtypes. Leukemia.

[127] Sehn L.H., Herrera A.F., Flowers C.R., Kamdar M.K., McMillan A., Hertzberg M., Assouline S., Kim T.M., Kim W.S., Ozcan M. (2020). Polatuzumab Vedotin in Relapsed or Refractory Diffuse Large B-Cell Lymphoma. J. Clin. Oncol..

[128] Flowers C.R., Matasar M.J., Herrera A.F., Hertzberg M., Assouline S., Demeter J., McMillan A., Mehta A., Opat S., Trněný M. (2024). Polatuzumab Vedotin plus Bendamustine and Rituximab or Obinutuzumab in Relapsed/Refractory Follicular Lymphoma: A Phase Ib/II Study. Haematologica.

[129] Tilly H., Morschhauser F., Sehn L.H., Friedberg J.W., Trněný M., Sharman J.P., Herbaux C., Burke J.M., Matasar M., Rai S. (2023). Polatuzumab Vedotin for the Front-Line Treatment of Diffuse Large B-Cell Lymphoma: A New Standard of Care?. J. Adv. Pract. Oncol..

[130] Tilly H., Morschhauser F., Sehn L.H., Friedberg J.W., Trněný M., Sharman J.P., Herbaux C., Burke J.M., Matasar M., Rai S. (2022). Polatuzumab Vedotin in Previously Untreated Diffuse Large B-Cell Lymphoma. N. Engl. J. Med..

[131] Sarraf Yazdy M., Kasamon Y.L., Gu W., Rodriguez L.R., Jin S., Bhatnagar V., Richardson N.C., Theoret M.R., Pazdur R., Gormley N.J. (2024). FDA Approval Summary: Polatuzumab Vedotin in the First-Line Treatment of Select Large B-Cell Lymphomas. Clin. Cancer Res..

[132] Markham A. (2020). Belantamab Mafodotin: First Approval. Drugs.

[133] Nooka A.K., Cohen A., Lee H.C., Badros A.Z., Suvannasankha A., Callander N., Abdallah A.-O., Trudel S., Chari A., Libby E. (2022). Single-Agent Belantamab Mafodotin in Patients with Relapsed or Refractory Multiple Myeloma: Final Analysis of the DREAMM-2 Trial. Blood.

[134] Dimopoulos M.A., Hungria V.T.M., Radinoff A., Delimpasi S., Mikala G., Masszi T., Li J., Capra M., Maiolino A., Pappa V. (2023). Efficacy and Safety of Single-Agent Belantamab Mafodotin versus Pomalidomide plus Low-Dose Dexamethasone in Patients with Relapsed or Refractory Multiple Myeloma (DREAMM-3): A Phase 3, Open-Label, Randomised Study. Lancet Haematol..

[135] Mateos M.-V., Robak P., Hus M., Xia Z., Zherebtsova V., Ward C., Ho P.J., Hajek R., Kim K., Dimopoulos M.A. (2024). Results from the Randomized Phase III DREAMM-7 Study of Belantamab Mafodotin (Belamaf) + Bortezomib, and Dexamethasone (BVd) vs Daratumumab, Bortezomib, and Dexamethasone (DVd) in Relapsed/Refractory Multiple Myeloma (RRMM). J. Clin. Oncol..

[136] Dimopoulos M.A., Beksac M., Pour L., Delimpasi S., Vorobyev V., Quach H., Spicka I., Radocha J., Robak P., Kim K. (2024). Belantamab Mafodotin, Pomalidomide, and Dexamethasone in Multiple Myeloma. N. Engl. J. Med..

[137] Morè S., Offidani M., Corvatta L., Petrucci M.T., Fazio F. (2023). Belantamab Mafodotin: From Clinical Trials Data to Real-Life Experiences. Cancers.

[138] Lee A. (2021). Loncastuximab Tesirine: First Approval. Drugs.

[139] Hamadani M., Radford J., Carlo-Stella C., Caimi P.F., Reid E., O’Connor O.A., Feingold J.M., Ardeshna K.M., Townsend W., Solh M. (2021). Final Results of a Phase 1 Study of Loncastuximab Tesirine in Relapsed/Refractory B-Cell Non-Hodgkin Lymphoma. Blood.

[140] Caimi P.F., Ai W., Alderuccio J.P., Ardeshna K.M., Hamadani M., Hess B., Kahl B.S., Radford J., Solh M., Stathis A. (2021). Loncastuximab Tesirine in Relapsed or Refractory Diffuse Large B-Cell Lymphoma (LOTIS-2): A Multicentre, Open-Label, Single-Arm, Phase 2 Trial. Lancet Oncol..

[141] Markham A., Al-Salama Z.T. (2022). Loncastuximab Tesirine in Relapsed or Refractory Diffuse Large B-Cell Lymphoma: A Profile of Its Use in the USA. Drugs Ther. Perspect..

[142] Wei S.C., Duffy C.R., Allison J.P. (2018). Fundamental Mechanisms of Immune Checkpoint Blockade Therapy. Cancer Discov..

[143] Chen L., Flies D.B. (2013). Molecular Mechanisms of T Cell Co-Stimulation and Co-Inhibition. Nat. Rev. Immunol..

[144] He X., Xu C. (2020). Immune Checkpoint Signaling and Cancer Immunotherapy. Cell Res..

[145] Iranzo P., Callejo A., Assaf J.D., Molina G., Lopez D.E., Garcia-Illescas D., Pardo N., Navarro A., Martinez-Marti A., Cedres S. (2022). Overview of Checkpoint Inhibitors Mechanism of Action: Role of Immune-Related Adverse Events and Their Treatment on Progression of Underlying Cancer. Front. Med..

[146] Hargadon K.M., Johnson C.E., Williams C.J. (2018). Immune Checkpoint Blockade Therapy for Cancer: An Overview of FDA-Approved Immune Checkpoint Inhibitors. Int. Immunopharmacol..

[147] Petitprez F., Meylan M., de Reyniès A., Sautès-Fridman C., Fridman W.H. (2020). The Tumor Microenvironment in the Response to Immune Checkpoint Blockade Therapies. Front. Immunol..

[148] Green M.R., Monti S., Rodig S.J., Juszczynski P., Currie T., O’Donnell E., Chapuy B., Takeyama K., Neuberg D., Golub T.R. (2010). Integrative Analysis Reveals Selective 9p24.1 Amplification, Increased PD-1 Ligand Expression, and Further Induction via JAK2 in Nodular Sclerosing Hodgkin Lymphoma and Primary Mediastinal Large B-Cell Lymphoma. Blood.

[149] Pianko M.J., Moskowitz A.J., Lesokhin A.M. (2018). Immunotherapy of Lymphoma and Myeloma: Facts and Hopes. Clin. Cancer Res..

[150] Armand P., Engert A., Younes A., Fanale M., Santoro A., Zinzani P.L., Timmerman J.M., Collins G.P., Ramchandren R., Cohen J.B. (2018). Nivolumab for Relapsed/Refractory Classic Hodgkin Lymphoma After Failure of Autologous Hematopoietic Cell Transplantation: Extended Follow-Up of the Multicohort Single-Arm Phase II CheckMate 205 Trial. J. Clin. Oncol..

[151] Ansell S.M., Bröckelmann P.J., von Keudell G., Lee H.J., Santoro A., Zinzani P.L., Collins G.P., Cohen J.B., de Boer J.P., Kuruvilla J. (2023). Nivolumab for Relapsed/Refractory Classical Hodgkin Lymphoma: 5-Year Survival from the Pivotal Phase 2 CheckMate 205 Study. Blood Adv..

[152] Younes A., Santoro A., Shipp M., Zinzani P.L., Timmerman J.M., Ansell S., Armand P., Fanale M., Ratanatharathorn V., Kuruvilla J. (2016). Nivolumab for Classical Hodgkin’s Lymphoma after Failure of Both Autologous Stem-Cell Transplantation and Brentuximab Vedotin: A Multicentre, Multicohort, Single-Arm Phase 2 Trial. Lancet. Oncol..

[153] Kasamon Y.L., Claro R.A.d., Wang Y., Shen Y.L., Farrell A.T., Pazdur R. (2017). FDA Approval Summary: Nivolumab for the Treatment of Relapsed or Progressive Classical Hodgkin Lymphoma. Oncologist.

[154] Herrera A.F., LeBlanc M., Castellino S.M., Li H., Rutherford S.C., Evens A.M., Davison K., Punnett A., Parsons S.K., Ahmed S. (2024). Nivolumab+AVD in Advanced-Stage Classic Hodgkin’s Lymphoma. N. Engl. J. Med..

[155] Armand P., Shipp M.A., Ribrag V., Michot J.M., Zinzani P.L., Kuruvilla J., Snyder E.S., Ricart A.D., Balakumaran A., Rose S. (2016). Programmed Death-1 Blockade with Pembrolizumab in Patients with Classical Hodgkin Lymphoma after Brentuximab Vedotin Failure. J. Clin. Oncol..

[156] Chen R., Zinzani P.L., Fanale M.A., Armand P., Johnson N.A., Brice P., Radford J., Ribrag V., Molin D., Vassilakopoulos T.P. (2017). Phase II Study of the Efficacy and Safety of Pembrolizumab for Relapsed/Refractory Classic Hodgkin Lymphoma. J. Clin. Oncol..

[157] Kuruvilla J., Ramchandren R., Santoro A., Paszkiewicz-Kozik E., Gasiorowski R., Johnson N.A., Fogliatto L.M., Goncalves I., de Oliveira J.S.R., Buccheri V. (2021). Pembrolizumab versus Brentuximab Vedotin in Relapsed or Refractory Classical Hodgkin Lymphoma (KEYNOTE-204): An Interim Analysis of a Multicentre, Randomised, Open-Label, Phase 3 Study. Lancet. Oncol..

[158] Voorhees T.J., Beaven A.W. (2020). Therapeutic Updates for Relapsed and Refractory Classical Hodgkin Lymphoma. Cancers.

[159] Armand P., Chen Y.B., Redd R.A., Joyce R.M., Bsat J., Jeter E., Merryman R.W., Coleman K.C., Dahi P.B., Nieto Y. (2019). PD-1 Blockade with Pembrolizumab for Classical Hodgkin Lymphoma after Autologous Stem Cell Transplantation. Blood.

[160] Mei M., Palmer J., Tsai N.-C., Lee H.J., Isufi I., Popplewell L.L., Smith L., Peters L., Rodriguez L., Godfrey J. (2022). Nivolumab Plus ICE As First Salvage Therapy in High-Risk Relapsed/Refractory Hodgkin Lymphoma. Blood.

[161] Mei M.G., Lee H.J., Palmer J.M., Chen R., Tsai N.C., Chen L., McBride K., Smith D.L., Melgar I., Song J.Y. (2022). Response-Adapted Anti-PD-1–Based Salvage Therapy for Hodgkin Lymphoma with Nivolumab Alone or in Combination with ICE. Blood.

[162] Zinzani P.L., Ribrag V., Moskowitz C.H., Michot J.M., Kuruvilla J., Balakumaran A., Zhang Y., Chlosta S., Shipp M.A., Armand P. (2017). Safety and Tolerability of Pembrolizumab in Patients with Relapsed/Refractory Primary Mediastinal Large B-Cell Lymphoma. Blood.

[163] Ding W., LaPlant B.R., Call T.G., Parikh S.A., Leis J.F., He R., Shanafelt T.D., Sinha S., Le-Rademacher J., Feldman A.L. (2017). Pembrolizumab in Patients with CLL and Richter Transformation or with Relapsed CLL. Blood.

[164] Melani C., Major A., Schowinsky J., Roschewski M., Pittaluga S., Jaffe E.S., Pack S.D., Abdullaev Z., Ahlman M.A., Kwak J.J. (2017). PD-1 Blockade in Mediastinal Gray-Zone Lymphoma. N. Engl. J. Med..

[165] Tomassetti S., Chen R., Dandapani S. (2019). The Role of Pembrolizumab in Relapsed/Refractory Primary Mediastinal Large B-Cell Lymphoma. Ther. Adv. Hematol..

[166] Jain N., Senapati J., Thakral B., Ferrajoli A., Thompson P., Burger J., Basu S., Kadia T., Daver N., Borthakur G. (2023). A Phase 2 Study of Nivolumab Combined with Ibrutinib in Patients with Diffuse Large B-Cell Richter Transformation of CLL. Blood Adv..

[167] Joshi M., Ansell S.M. (2020). Activating the Antitumor Immune Response in Non-Hodgkin Lymphoma Using Immune Checkpoint Inhibitors. J. Immunol. Res..

[168] Apostolidis J., Sayyed A., Darweesh M., Kaloyannidis P., Al Hashmi H. (2020). Current Clinical Applications and Future Perspectives of Immune Checkpoint Inhibitors in Non-Hodgkin Lymphoma. J. Immunol. Res..

[169] Chari A., Suvannasankha A., Fay J.W., Arnulf B., Kaufman J.L., Ifthikharuddin J.J., Weiss B.M., Krishnan A., Lentzsch S., Comenzo R. (2017). Daratumumab plus Pomalidomide and Dexamethasone in Relapsed and/or Refractory Multiple Myeloma. Blood.

[170] Mateos M.V., Blacklock H., Schjesvold F., Oriol A., Simpson D., George A., Goldschmidt H., Larocca A., Chanan-Khan A., Sherbenou D. (2019). Pembrolizumab plus Pomalidomide and Dexamethasone for Patients with Relapsed or Refractory Multiple Myeloma (KEYNOTE-183): A Randomised, Open-Label, Phase 3 Trial. Lancet. Haematol..

[171] Liu Z., Xu X., Liu H., Zhao X., Yang C., Fu R. (2023). Immune Checkpoint Inhibitors for Multiple Myeloma Immunotherapy. Exp. Hematol. Oncol..

[172] Mun J.Y., Leem S.H., Lee J.H., Kim H.S. (2022). Dual Relationship Between Stromal Cells and Immune Cells in the Tumor Microenvironment. Front. Immunol..

[173] Li Z.W., Dalton W.S. (2006). Tumor Microenvironment and Drug Resistance in Hematologic Malignancies. Blood Rev..

[174] Arandi N., Dehghani M. (2023). Immune Microenvironment in Hematologic Malignancies. Iran. J. Med. Sci..

[175] Kotlov N., Bagaev A., Revuelta M.V., Phillip J.M., Cacciapuoti M.T., Antysheva Z., Svekolkin V., Tikhonova E., Miheecheva N., Kuzkina N. (2021). Clinical and Biological Subtypes of B-Cell Lymphoma Revealed by Microenvironmental Signatures. Cancer Discov..

[176] Höpken U.E., Rehm A. (2019). Targeting the Tumor Microenvironment of Leukemia and Lymphoma. Trends Cancer.

[177] Bilotta M.T., Antignani A., Fitzgerald D.J. (2022). Managing the TME to Improve the Efficacy of Cancer Therapy. Front. Immunol..

[178] Buchbinder E.I., Desai A. (2016). CTLA-4 and PD-1 Pathways Similarities, Differences, and Implications of Their Inhibition. Am. J. Clin. Oncol. Cancer Clin. Trials.

[179] Sinkarevs S., Strumfs B., Volkova S., Strumfa I. (2024). Tumour Microenvironment: The General Principles of Pathogenesis and Implications in Diffuse Large B Cell Lymphoma. Cells.

[180] Mulder T.A., Wahlin B.E., Österborg A., Palma M. (2019). Targeting the Immune Microenvironment in Lymphomas of B-Cell Origin: From Biology to Clinical Application. Cancers.

[181] Li Y., Wu Y., Hu Y. (2021). Metabolites in the Tumor Microenvironment Reprogram Functions of Immune Effector Cells Through Epigenetic Modifications. Front. Immunol..

[182] Waldman A.D., Fritz J.M., Lenardo M.J. (2020). A Guide to Cancer Immunotherapy: From T Cell Basic Science to Clinical Practice. Nat. Rev. Immunol..

[183] Gulla A., Anderson K.C. (2020). Multiple Myeloma: The (r)Evolution of Current Therapy and a Glance into the Future. Haematologica.

[184] Patel K., Pagel J.M. (2021). Current and Future Treatment Strategies in Chronic Lymphocytic Leukemia. J. Hematol. Oncol..

[185] Hallek M., Al-Sawaf O. (2021). Chronic Lymphocytic Leukemia: 2022 Update on Diagnostic and Therapeutic Procedures. Am. J. Hematol..

[186] Tavarozzi R., Zacchi G., Pietrasanta D., Catania G., Castellino A., Monaco F., Gandolfo C., Rivela P., Sofia A., Schiena N. (2023). Changing Trends in B-Cell Non-Hodgkin Lymphoma Treatment: The Role of Novel Monoclonal Antibodies in Clinical Practice. Cancers.

[187] Shepard H.M., Phillips G.L., Thanos C.D., Feldmann M. (2017). Developments in Therapy with Monoclonal Antibodies and Related Proteins. Clin. Med..

[188] Chiu M.L., Goulet D.R., Teplyakov A., Gilliland G.L. (2019). Antibody Structure and Function: The Basis for Engineering Therapeutics. Antibodies.

[189] Scott A.M., Wolchok J.D., Old L.J. (2012). Antibody Therapy of Cancer. Nat. Rev. Cancer.

[190] Isabwe G.A.C., Garcia Neuer M., de las Vecillas Sanchez L., Lynch D.M., Marquis K., Castells M. (2018). Hypersensitivity Reactions to Therapeutic Monoclonal Antibodies: Phenotypes and Endotypes. J. Allergy Clin. Immunol..

[191] Gelis S., Verdesoto J.T., Pascal M., Muñoz-Cano R.M. (2022). Hypersensitivity Reactions to Monoclonal Antibodies: New Approaches. Curr. Treat. Options Allergy.

[192] Picard M., Galvão V.R. (2017). Current Knowledge and Management of Hypersensitivity Reactions to Monoclonal Antibodies. J. Allergy Clin. Immunol. Pract..

[193] Isabwe G.A.C., Sanchez L.D.L.V., Castells M. (2017). Management of Adverse Reactions to Biologic Agents. Allergy Asthma Proc..

[194] Pavanello F., Zucca E., Ghielmini M. (2017). Rituximab: 13 Open Questions after 20years of Clinical Use. Cancer Treat. Rev..

[195] Leipold D., Prabhu S. (2019). Pharmacokinetic and Pharmacodynamic Considerations in the Design of Therapeutic Antibodies. Clin. Transl. Sci..

[196] Wang Q., Chen Y., Park J., Liu X., Hu Y., Wang T., McFarland K., Betenbaugh M.J. (2019). Design and Production of Bispecific Antibodies. Antibodies.

[197] Jureczek J., Bergmann R., Berndt N., Koristka S., Kegler A., Puentes-Cala E., Soto J.A., Arndt C., Bachmann M., Feldmann A. (2019). An Oligo-His-Tag of a Targeting Module Does Not Influence Its Biodistribution and the Retargeting Capabilities of UniCAR T Cells. Sci. Rep..

[198] Strohl W.R. (2015). Fusion Proteins for Half-Life Extension of Biologics as a Strategy to Make Biobetters. BioDrugs.

[199] Jureczek J., Feldmann A., Bergmann R., Arndt C., Berndt N., Koristka S., Loureiro L.R., Mitwasi N., Hoffmann A., Kegler A. (2020). Highly Efficient Targeting of EGFR-Expressing Tumor Cells with UNiCAR T Cells via Target Modules Based on Cetuximab^®^. Onco. Targets. Ther..

[200] Elshiaty M., Schindler H., Christopoulos P. (2021). Principles and Current Clinical Landscape of Multispecific Antibodies against Cancer. Int. J. Mol. Sci..

[201] Surowka M., Klein C. (2024). A Pivotal Decade for Bispecific Antibodies?. MAbs.

[202] Cassanello G., Luna de Abia A., Falchi L. (2024). Trial Watch: Bispecific Antibodies for the Treatment of Relapsed or Refractory Large B-Cell Lymphoma. Oncoimmunology.

[203] Caraccio C., Krishna S., Phillips D.J., Schürch C.M. (2020). Bispecific Antibodies for Multiple Myeloma: A Review of Targets, Drugs, Clinical Trials, and Future Directions. Front. Immunol..

[204] Dickopf S., Georges G.J., Brinkmann U. (2020). Format and Geometries Matter: Structure-Based Design Defines the Functionality of Bispecific Antibodies. Comput. Struct. Biotechnol. J..

[205] Ma J., Mo Y., Tang M., Shen J., Qi Y., Zhao W., Huang Y., Xu Y., Qian C. (2021). Bispecific Antibodies: From Research to Clinical Application. Front. Immunol..

[206] Gerber H.P., Gangwar S., Betts A. (2023). Therapeutic Index Improvement of Antibody-Drug Conjugates. MAbs.

[207] Dean A.Q., Luo S., Twomey J.D., Zhang B. (2021). Targeting Cancer with Antibody-Drug Conjugates: Promises and Challenges. MAbs.

[208] Weggen J.T., Bean R., Hui K., Wendeler M., Hubbuch J. (2024). Kinetic Models towards an Enhanced Understanding of Diverse ADC Conjugation Reactions. Front. Bioeng. Biotechnol..

[209] Fuentes-Antrás J., Genta S., Vijenthira A., Siu L.L. (2023). Antibody-Drug Conjugates: In Search of Partners of Choice. Trends Cancer.

[210] Cuesta-Mateos C., Alcaraz-Serna A., Somovilla-Crespo B., Muñoz-Calleja C. (2018). Monoclonal Antibody Therapies for Hematological Malignancies: Not Just Lineage-Specific Targets. Front. Immunol..

[211] Molica M., Perrone S., Andriola C., Rossi M. (2023). Immunotherapy with Monoclonal Antibodies for Acute Myeloid Leukemia: A Work in Progress. Cancers.

[212] D’Alò F., Bellesi S., Maiolo E., Alma E., Bellisario F., Malafronte R., Viscovo M., Campana F., Hohaus S. (2024). Novel Targets and Advanced Therapies in Diffuse Large B Cell Lymphomas. Cancers.

[213] Crombie J.L., Graff T., Falchi L., Karimi Y.H., Bannerji R., Nastoupil L., Thieblemont C., Ursu R., Bartlett N., Nachar V. (2024). Consensus Recommendations on the Management of Toxicity Associated with CD3×CD20 Bispecific Antibody Therapy. Blood.

[214] Madsen A.V., Pedersen L.E., Kristensen P., Goletz S. (2024). Design and Engineering of Bispecific Antibodies: Insights and Practical Considerations. Front. Bioeng. Biotechnol..

